# Differential transcription pathways associated with rootstock-induced dwarfing in breadfruit (*Artocarpus altilis*) scions

**DOI:** 10.1186/s12870-021-03013-6

**Published:** 2021-06-05

**Authors:** Yuchan Zhou, Steven J. R. Underhill

**Affiliations:** 1 Australian Centre for Pacific Islands Research, University of the Sunshine Coast, Sippy Downs, QLD 4556 Australia; 2 Queensland Alliance for Agriculture and Food Innovation, University of Queensland, St Lucia, QLD 4072 Australia

**Keywords:** Breadfruit (*Artocarpus altilis*), Marang (*Artocarpus odoratissimus*), Dwarfing, Rootstock, Stem elongation, RNA-sequencing, Transcriptome, De novo assembly

## Abstract

**Background:**

Breadfruit (*Artocarpus altilis*) is a traditional staple tree crop throughout the tropics. Through interspecific grafting, a dwarf phenotype with over 50% reduction in plant height was identified when marang (*Artocarpus odoratissimus*) rootstocks were used. However, the molecular mechanism underlying the rootstock-induced breadfruit dwarfing is poorly understood.

**Results:**

An RNA-sequencing study of breadfruit scions at 22 months after grafting identified 5409 differentially expressed genes (DEGs) of which 2069 were upregulated and 3339 were downregulated in scion stems on marang rootstocks compared to those on self-graft. The DEGs were predominantly enriched for biological processes involved in carbon metabolism, cell wall organization, plant hormone signal transduction and redox homeostasis. The down-regulation of genes encoding vacuolar acid invertases and alkaline/neutral invertases, was consistent with the decreased activity of both enzymes, accompanying with a higher sucrose but lower glucose and fructose levels in the tissues. Key genes of biosynthetic pathways for amino acids, lipids and cell wall were down regulated, reflecting reduction of sucrose utilisation for stem growth on dwarfing rootstocks. Genes encoding sugar transporters, amino acid transporters, choline transporters, along with large number of potassium channels and aquaporin family members were down-regulated in scion stems on marang rootstocks. Lower activity of plasma membrane H^+^-ATPase, together with the predominance of genes encoding expansins, wall-associated receptor kinases and key enzymes for biosynthesis and re-modelling of cellulose, xyloglucans and pectins in down-regulated DGEs suggested impairment of cell expansion. Signalling pathways of auxin and gibberellin, along with strigolacton and brassinosteroid biosynthetic genes dominated the down-regulated DEGs. Phenylpropanoid pathway was enriched, with key lignin biosynthetic genes down-regulated, and flavonoid biosynthetic genes upregulated in scions on marang rootstocks. Signalling pathways of salicylic acid, jasmonic acid, ethylene and MAPK cascade were significantly enriched in the upregulated DEGs.

**Conclusions:**

Rootstock-induced disruption in pathways regulating nutrient transport, sucrose utilisation, cell wall biosynthesis and networks of hormone transduction are proposed to impair cell expansion and stem elongation, leading to dwarf phenotype in breadfruit scions. The information provides opportunity to develop screening strategy for rootstock breeding and selection for breadfruit dwarfing.

**Supplementary Information:**

The online version contains supplementary material available at 10.1186/s12870-021-03013-6.

## Background

Trees with reduced stature facilitate high-density planting, tree management and harvesting. In temperate fruit tree cultivation, tree dwarfing has been achieved through the widespread use of dwarfing rootstocks. Despite the importance of rootstock-induced dwarfing, the underlying mechanism of the phenomenon is not well understood. Breadfruit [*Artocarpus altilis* (Parkinson) Fosberg)] is a tropical tree crop from 15 to 20 m. The species is principally grown as an energy food, a source of complex carbohydrates, vitamins and minerals, and is regarded as a food security crop in the tropics [1]. Transition toward high-density commercial planting, as well as tree loss due to intense tropical windstorm in the regions has driven an interest in small size breadfruit trees [2]. Breadfruit has hundreds of cultivars with great diversity in morphological and agronomic characteristics [1], but the dwarf variety of the species has not been reported. Through interspecific grafting, rootstock of marang (*Artocarpus odoratissimus* Blanco) was identified as a size-controlling rootstock conferring over 50% reduction in breadfruit scion size, with over 70% shorter internode length compared to those on self-graft and the standard, non-grafted breadfruit plants [3, 4]. Breadfruit plants on marang rootstocks grows normally except for being dwarf, providing a potential solution for tree vigour control in commercial cultivation [3]. Under the same genus of *Artocarpus*, marang is also a large tropical fruit tree to 25 m, no dwarf phenotype has been identified. Little is known about the intriguing interactive processes by which marang greatly reduces the tree size of its grafted scions when used as rootstocks.

Several physiological mechanisms have been proposed to explain rootstock-induced dwarfing. First, the anatomical change surrounding graft union has been assumed to act as a barrier for movement of nutrients, hormones or other signals between rootstock and scion [5]. Reduction in solute and water transport across graft union has been implicated as causal factors for rootstock-induced dwarfing [6, 7]. The hydraulic conductance was shown to increase with the vigour of apple rootstocks [7]. The reduction in stem extension rate of peach trees was correlated to the decreased water potential in stems of dwarfing rootstocks [6]. Graft union of dwarfing cherry rootstocks has been shown to limit soluble sugar transport or starch mobilization, leading to decrease in vegetative growth [8]. Another hypothesis includes the altered hormone signalling occurred between scions and rootstocks [9, 10]. Reduction in auxin transport from aerial parts of grafted plants has been proposed to limit root growth and cytokinin production supplied for shoot growth [9]. Dwarfing apple rootstocks have been shown to limit root-produced gibberellic acid (GA) precursor, GA_19_ to scions [10–12], as a result, application of GA to apple scions on dwarfing rootstocks restores the node number of both the primary axis and secondary shoots [13]. Previously exogenous GA treatment was also shown to restore the stem elongation rate and the internode length of breadfruit scions growing on marang rootstocks [4]. On the other hand, abscisic acid (ABA) concentration is higher in scion tissues on apple dwarfing rootstocks [10]. Over-accumulation of flavonoids has also been suggested to affect auxin level and scion growth on dwarfing apple rootstocks [9, 14]. Several of these mechanisms have been tested many times, inconsistent results have also been shown [15].

As a first step toward understanding the molecular mechanism underlying breadfruit dwarfing induced by marang rootstocks, we undertook an RNA-sequencing study in combination with comparative biochemical analysis to identify biological processes involved in the phenotype variation of breadfruit scions growing on different rootstocks. To better characterize differential gene expression associated with rootstock response, scion stem transcriptome was de novo assembled and transcriptome comparison was used to identify gene cohorts and regulatory pathways associated with stem elongation growth. The work provides insight into the signal transduction pathways regulating breadfruit dwarfing by interspecific rootstocks, also generates a valuable gene resource for functional study and rootstock breeding for dwarfism of the species.

## Results

### Effect of rootstocks on growth characteristics of breadfruit scions

**Fig. 1:**
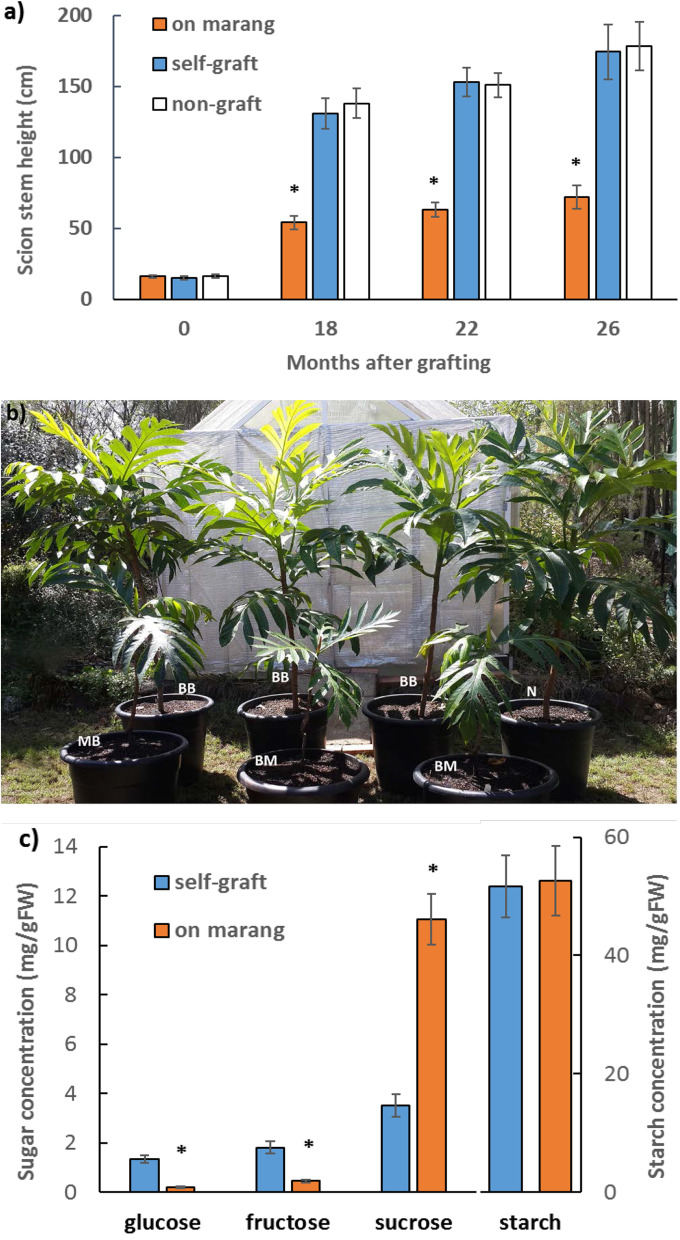
Rootstock effect on the phenotype of breadfruit scions. **a** Height of breadfruit scion main stems growing on different rootstocks in the period up to 26 months after grafting. **b** Representatives of breadfruit plants growing on different rootstocks at 22 months after grafting. BM, breadfruit plants on marang rootstocks; BB, self-graft; N, non-graft. **c** Non-structural carbohydrate contents of scion stems on different rootstocks at 22 months after grafting. All values represent mean ± SE of five biological replicates (* *p* < 0.05)

Breadfruit plants on marang rootstocks displayed significantly shorter stature than the standard non-grafted plants as well as the self-grafted plants, with about 58% reduction in scion stem height compared to those on self-graft in the period from 18 to 26 months after grafting (Fig. 1a and b). There was no difference in the growth habit between the self-graft and the non-graft. Except for being dwarf, breadfruit plants on marang rootstocks grew normally under current glasshouse condition. Same as those of the self-graft, all these plants displayed progressive development of new internodes from apical buds or axillary buds and continuous emergence of green leaves from the apex (see example image in Figure S1). Compared with the growth parameters from 3 to 18 months after grafting [3], growth analysis at 26 months (Table S1) showed plants of both graft types had significant growth with increase in scion height, stem diameter and node number. However, plants on marang rootstocks continued to display shorter stature and reduced stem thickness, but no change in stem node number. Although with smaller leaves, these plants showed no change in other leaf characters, such as leaf colour and leaf shape (Fig. 1; Figure S1). As the performance of a grafted plant and the commercial application of the rootstock not only depends on the genotype of scion and rootstock, but also the compatibility of the rootstock with scion [16–18], it is necessary to examine the graft combination of breadfruit/marang for graft compatibility. Anatomy examination of a scion/rootstock union has been a method that unambiguously identifies graft incompatibility in woody species [19–21]. Anatomy examination of graft interface was first carried out on the graft unions of three interspecific grafts at 26 months after grafting. The results revealed that the breadfruit/marang grafts had good compatibility with hardly visible graft lines in bark and wood (category ‘A’, see Methods, also [22]) and there was no discontinuities along the graft lines between bark and wood in all the graft unions examined (see example image in Figure S2). The ratio of the stem diameter above and below the graft union has often been used to correlate growth characteristics with graft incompatibility [19–21]. In consistent with the anatomy assessment, the ratio of scion to rootstock stem diameter in the breadfruit/marang combination was very close to 1, and there was no significant difference between those of the self-graft and the interspecific graft (Table S1). Scion leaf chlorophyll (Chl) concentration, reflecting the capability of carbon assimilation and proper functioning of a composite grafted plant, has also served as a non-destructive tool to identify graft incompatibility [19–21]. Here, concentration of scion leaf chlorophyll on marang rootstocks, including Chl *a*, Chl *b*, Chl *a* + Chl *b*, and Chl *a/b* was not significantly different from those of the self-graft (Table S1). Consistent with the results, a previous monthly measurement of chlorophyll by a non-destructive method also showed no difference in total chlorophyll of scion leaves on different rootstocks at any time point [3]. Furthermore, there were no difference in the 1-year and 2-year compatibility rates between the interspecific graft and the self-graft (Figure S1). Taken together, these evidences confirmed that the grafts of breadfruit/marang combination had good compatibility in the period up to 26 months after grafting. The dwarf phenotype of breadfruit scions on marang rootstocks was therefore not associated with graft incompatibility.

Non-structural carbohydrate contents (NSC) were further measured at 22 months after grafting (Fig. 1c). Scion stems on marang rootstocks showed significantly lower levels of glucose and fructose, but higher levels of sucrose, with 84.5% reduction in glucose and 74.7% reduction in fructose, but over 214% increase in sucrose compared to those on self-graft. There was no significant difference in starch content across all samples (Fig. 1c).

### Breadfruit scion stem transcriptome analysis

First, a transcriptome library was de novo assembled for breadfruit scion stems. It included a total of 520.6 million paired-end reads (2 × 150 bp) generated from sequencing across all samples. After filtering raw reads, a total of 516.2 million clean paired-reads were produced, with 95.0% having a Phred quality score of ≥ Q20. All clean reads were de novo assembled into 212,878 contigs (> 297 bp), with N50 of 2928 bp and 96.8% complete Benchmarking Universal Single-Copy Orthologs (BUSCO) in the final transcriptome assembly (Table S2). The unigenes were annotated by query against various public databases (see Methods). As a result, 77,390 (61.41%) unigenes were matched to one or more of these databases. The assembled unigene sequences are available at NCBI BioProject database (https://www.ncbi.nlm.nih.gov/bioproject/) with BioProject ID: PRJNA679862.

**Fig. 2:**
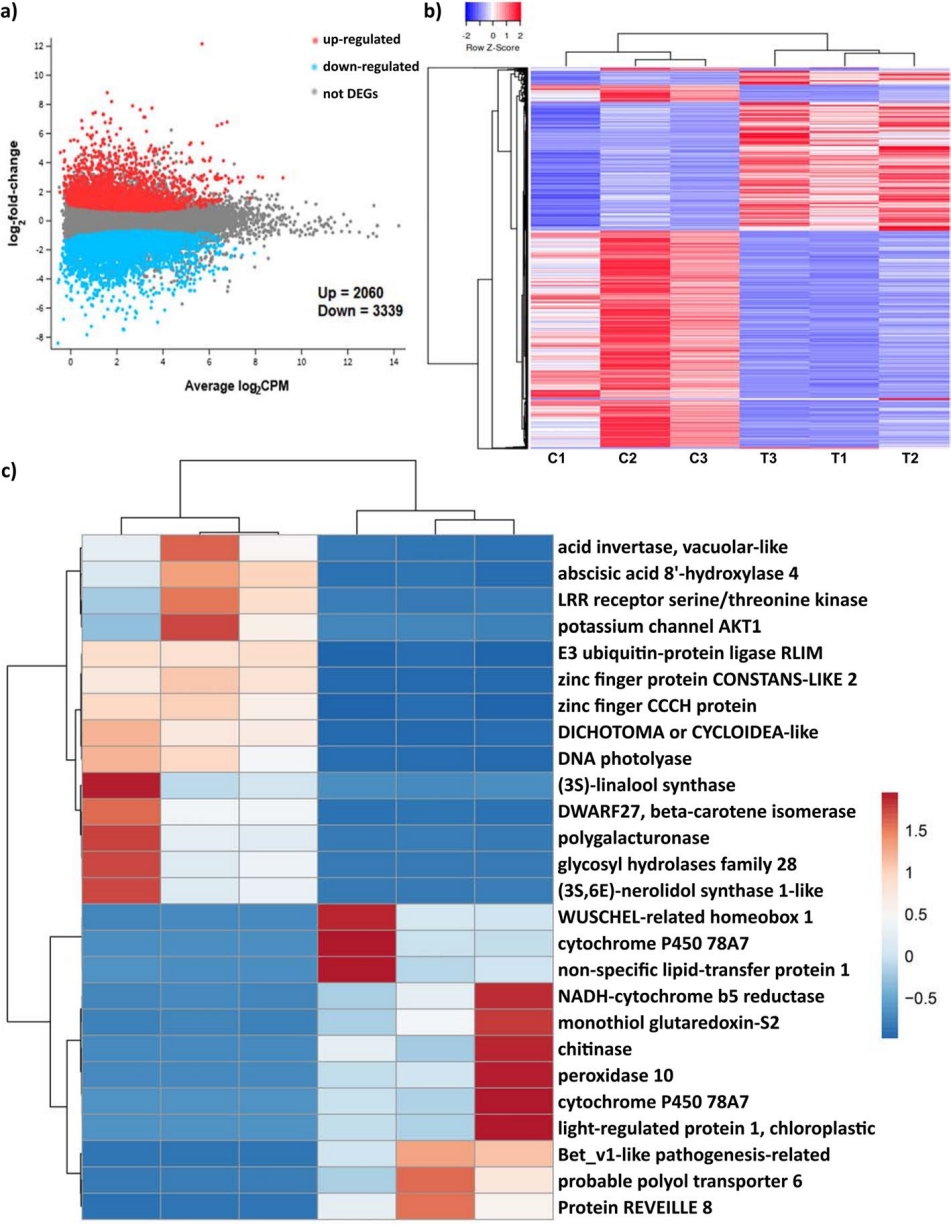
Rootstock effect on global gene expression pattern revealed by RNA-sequencing. **a** Scatter plots comparing all expressed genes of scion stems on marang rootstocks vs. those on self-graft. Differentially expressed genes (FDR < 0.05 and |log_2_FC|≥ 1) are donated in red or blue. **b** Heatmap and hierarchical clustering of differentially expressed genes among biologic replicates of graft combination. Heatmap was constructed with Heatmapper (http://www.heatmapper.ca/). Values of gene expression level are converted to Z-Score. C1-C3, replicates of self-graft (control); T1-T3, replicates of breadfruits on marang rootstocks. **c** Heatmap and hierarchical clustering of top major DEGs constructed by ClustVis (https://biit.cs.ut.ee/clustvis/). Colour codes represent standardized gene expression levels. Euclidean distance and Ward linkage were used to construct dendrograms against genes and samples. DEGs, differentially expressed genes

To identify global gene expression patterns related to rootstock-induced dwarfing, total RNA from the second internodes growing on different rootstocks at 22 months after grafting, was processed for RNA-sequencing (RNA-seq) on an Illumina platform (see Methods). Between 32 to 45 million clean paired-end reads were generated for each biological replicate, with 92.9% to 95.5% mapped back to the assembled transcriptome (Table S2). Using the cut-off of adjusted *p* < 0.05 & |log_2_ fold change|≥ 1, a total of 5409 differentially expressed genes (DEGs) were identified, including 2069 upregulated and 3339 downregulated genes in scion stems growing on marang rootstocks compared to those on self-graft (Fig. 2). The top major DEGs included up-regulated genes encoding Bet_v1-like pathogenesis-related protein, peroxidase 10, chitinase and WUSCHEL-related homeobox 1, all of which had log_2_FC > 6, and down-regulated genes encoding vacuolar-like acid invertase, potassium channel AKT1, beta-carotene isomerase (DWARF27) and polygalacturonase, all of which had Log_2_FC < -5 (Fig. 2c).

**Fig. 3:**
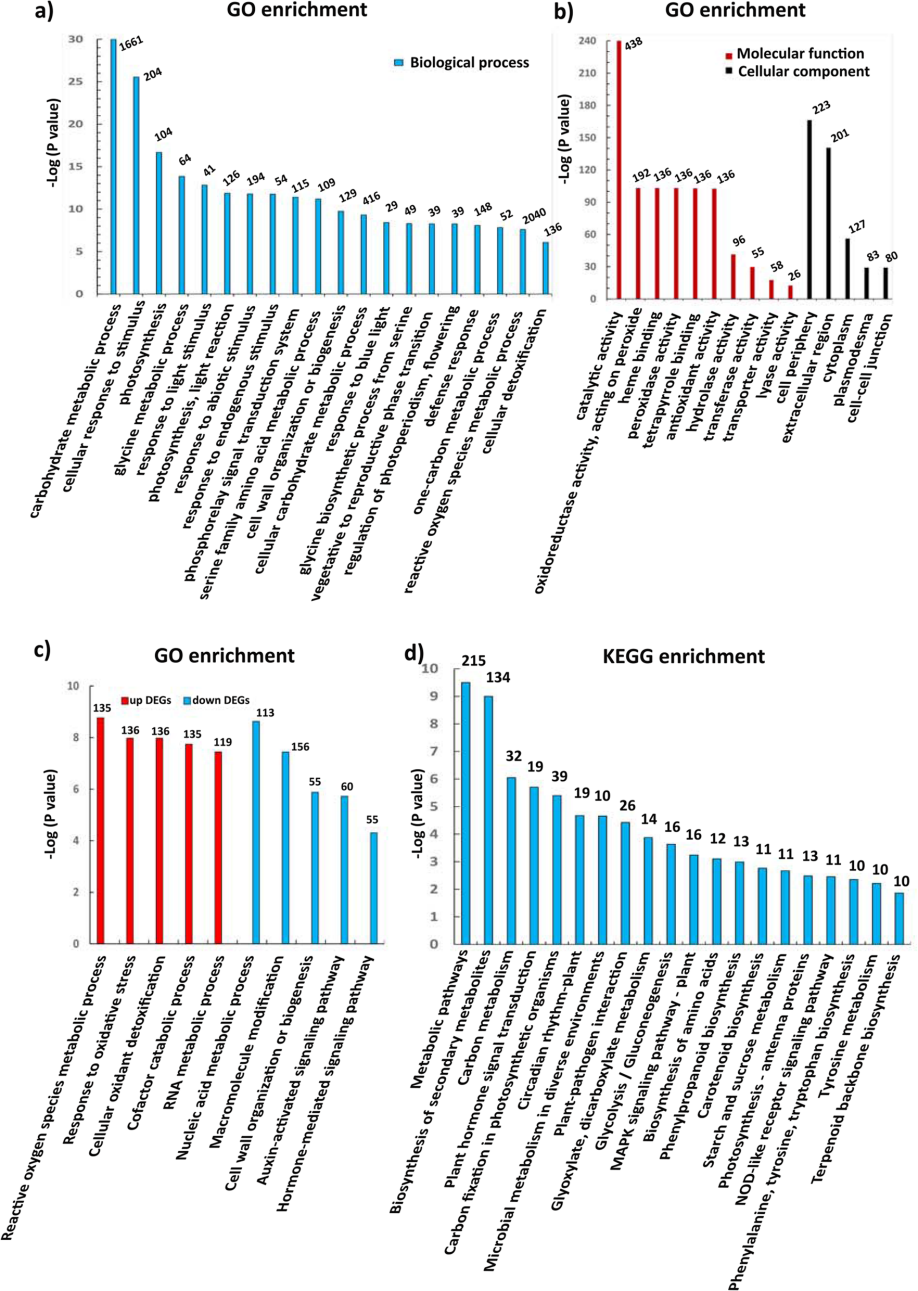
GO and KEGG enrichment analysis of differentially expressed genes. **a** Top 20 enriched GO terms in biological process; **b** Enriched GO terms in molecular function and cellular component; **c** Enriched GO terms in biological process among up- and down-regulated genes; **d** Top 20 enriched KEGG pathways. The number of genes enriched is displayed on top of each bar graph. DEGs, differentially expressed genes

Analysis of gene ontology (GO) and Kyoto Encyclopedia of Genes and Genomes (KEGG) enrichment was performed for DEGs. In GO analysis, all DEGs were annotated with GO terms and categorized into three ontologies: biological processes, molecular functions and cellular components. Of the top 20 enriched GO terms (Fig. 3), the carbohydrate metabolic process and cellular response to stimulus were the most enriched biological processes. Biological processes regulating vegetative growth were highly enriched, including response to blue light and cell wall organization or biogenesis. Cell protective processes, such as phosphorelay signal transduction system, defense response and cellular detoxification were also enriched (Fig. 3a). DEGs were predominantly enriched for molecular function associated with oxidoreductase activity on peroxide, peroxidase activity and antioxidant activity, with cell periphery and extracellular region being the main cellular components (Fig. 3b). GO enrichment was further analyzed among the up- or down-regulated DEGs. These revealed biological processes related to reactive oxygen species (ROS) metabolic process, response to oxidative stress and cellular oxidant detoxification were the most enriched GO terms in the upregulated genes, and biological processes participating in nucleic acid metabolic process, macromolecule modification and cell wall organization or biogenesis were predominantly enriched in the down-regulated genes (Fig. 3c). Among the top 20 enriched KEGG pathways were biosynthesis of secondary metabolites, carbon metabolism and plant hormone signal transduction (Fig. 3d). The highly enriched pathways also included plant-pathogen interaction, MAPK signalling pathway, phenylpropanoid biosynthesis and NOD-like receptor signalling pathway, consistent with the enriched GO terms associated with cell protective processes (Fig. 3).

### Genes involved in nutrient transport and primary metabolism

**Fig. 4:**
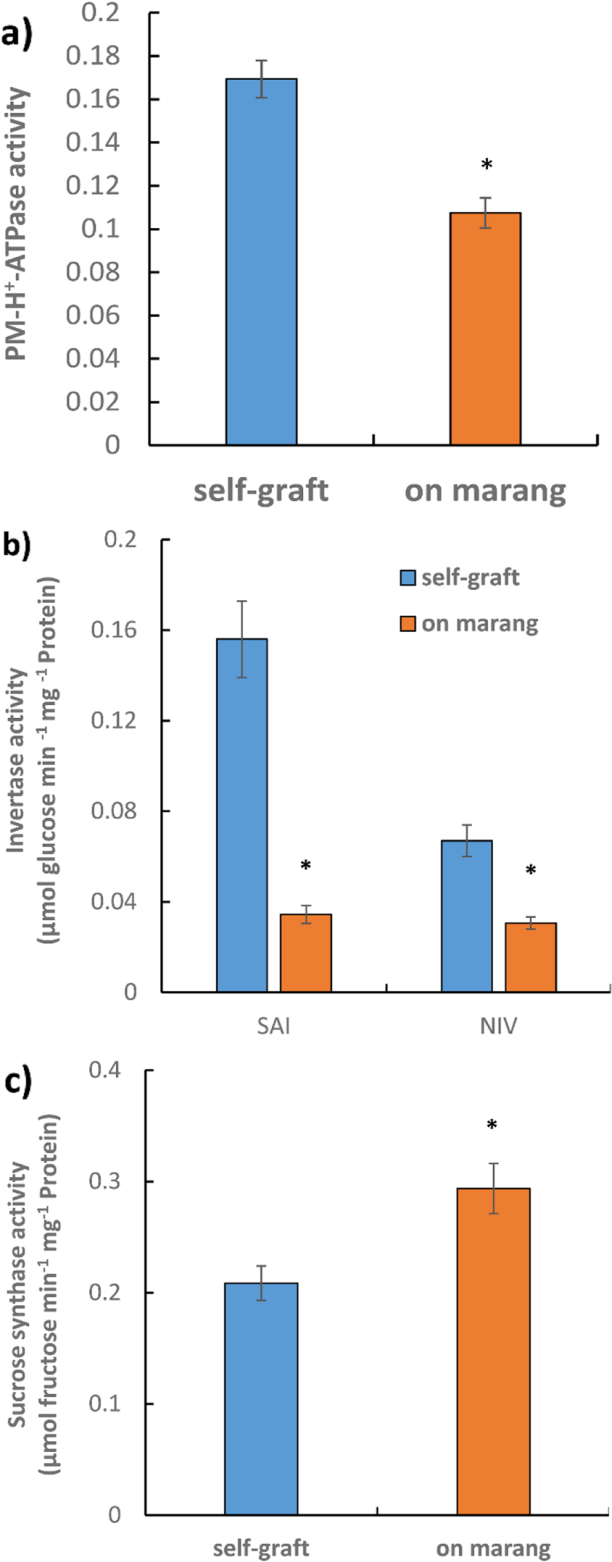
Activity of plasma membrane H^+^-ATPase and sucrose-degradation enzymes of breadfruit scion stems on different rootstocks at 22 months after grafting. The activity of plasma membrane H^+^-ATPase, represented as the vanadate-sensitive ATPase, was calculated from the difference between activities in the absence and presence of 1 mM Na_3_VO_4_. PM H^+^-ATPase, plasma membrane H + -ATPase; SAI, soluble acid invertase; NIV, neutral invertase. All values represent mean ± SE of three biological replicates (* *p* < 0.05)

Membrane transporters play pivotal roles in facilitating translocation of ions and solutes in plants, and their activities impact carbon allocation and plant growth [23]. A total of 161 out of 246 DEGs identified as member transporter genes were down regulated in stems growing on marang rootstocks compared to those on self-graft (Table S3, Table 1). These included genes encoding sucrose/H^+^ symporter SUT1, tonoplast‐localized SUT4-like, SWEETs, choline transporter-like, along with large numbers of amino acid transporters (8 DEGs), tonoplast dicarboxylate transporters (11 DEGs), aquaporin/major intrinsic protein family (26 DEGs) and potassium channels (AKT1, 5 DEGs; AKT2/3, 4 DEGs; potassium channel SKOR, 11 DEGs, Table S3). High number of genes encoding plasma membrane (PM) H^+^-ATPase was down-regulated (13 out of 18 DEGs, Table S3). Lower activity of PM H^+^-ATPase was previously found in scion stems growing on marang rootstocks at 12 and 18 months after grafting [3], decreased activity of the enzyme was also found at 22 months, with 36.6% reduction compared to that of the self-graft (Fig. 4a).

**Table 1:** Genes involved in nutrient transport and metabolism of carbohydrates, amino acids and lipids

Description	Expression pattern	Gene ID
***Nutrient transport***		
Sucrose/H^+^ symporter SUT1	down	trinity_40896
Tonoplast‐localized sucrose transport SUT4-like	down	rnaspades_39330
Sugar (and other) transporter	down	oases_192581, oases_137976, oases_192586
Proton-dependent oligopeptide transporter	down	oases_213155, trinity_32858
Potassium channel AKT1	down	oases_475576, oases_475593, oases_475606,
		oases_475614, trinity_15808
Potassium channel AKT2/3	down	oases_380431, oases_475578, oases_475619, oases_475589
SWEET sugar transporter	down	trinity_59504, trinity_59506; oases_190576; oases_190572
Choline transporter-like	down	oases_181685, oases_181691, oases_181688, oases_181689
***Carbohydrate metabolism***		
Granule-bound starch synthase	down	oases_169727, oases_169703, rnaspades_98532
α-amylase	up	oases_552865, oases_552874, oases_335134
Starch phosphorylase	up	trinity_296064
***Amino acid metabolism***		
Methyltetrahydropteroyltriglutamate-	down	oases_261494
Homocysteine methyltransferase; metE		
Threonine ammonia-lyase; ilvA	down	trinity_346450, trinity_346451, rnaspades_61830
serB; phosphoserine phosphatase	down	oases_329556, oases_261902
N-acetylglutamate synthase (NAGS)	down	rnaspades_30298
Serine hydroxymethyltransferase; glyA	down	trinity_26220, oases_123058, oases_123030
		oases_123034, rnaspades_49844
6-phosphofructokinase 1; pfkA	down	oases_173227
Pyruvate kinase	down	oases_329556
Shikimate kinase; aroK	down	trinity_74402
3-deoxy-7-phosphoheptulonate synthase	down	trinity_190957, oases_280413, oases_280417,
(DAHP synthase)		trinity_190966, trinity_190974
3-dehydroquinate dehydratase (DHQase)	down	oases_251725, oases_251713
Chorismate synthase; aroC	down	oases_255980, rnaspades_8947
Ornithine carbamoyltransferase	up	oases_175970
NADP-specific glutamate dehydrogenase	up	oases_549773
Branched-chain amino acid aminotransferase	up	trinity_70257
Fumarylacetoacetase	up	oases_73908
***Lipid metabolism***		
3-oxoacyl- synthase II; fabF	down	oases_404351
Fatty acyl-ACP thioesterase B; FATB	down	oases_139817
Acyl- desaturase; FAB2	down	oases_83116
Acyl-coenzyme A thioesterase	down	oases_238563
ß -ketoacyl synthase / very-long-chain 3-oxoacyl-CoA reductase	down	trinity_169921
Very-long-chain (3R)-3-hydroxyacyl-CoA dehydratase	down	oases_336848, trinity_284802
Enoyl-CoA hydratase/3-hydroxyacyl-CoA dehydrogenase	up	oases_148807, oases_290131

In sucrose metabolism, a total of 29 DEGs identified as invertase genes, including genes for vacuolar acid invertases (SAIs, 20 DEGs) and alkaline /neutral invertases (NIVs, 9 DEGs), were down-regulated, but 10 DEGs encoding sucrose synthase (SUSY) were up regulated in scion stems on marang rootstocks (Table S3). There were no change in the expression of sucrose phosphate synthase genes (Table S3). Consistently, significantly lower activity of SAI and NIV, but higher activity of SUSY was found in these tissues, with 77.9% reduction for SAI, 54.2% reduction for NIV but 40.8% increase for SUSY compared to that of the self-graft (Fig. 4b).

Key genes for biosynthesis of starch, amino acid and fatty acids were down regulated in scion stems on marang rootstocks (Table 1, Table S3). These included genes for starch synthase (8 DEGs, Table S3) and granule-bound starch synthase in starch biosynthesis; genes for 5-methyltetrahydropteroyltriglutamate–homocysteine methyltransferase (metE), serine hydroxymethyltransferase and Shikimate pathway (DAHP synthase, 3-DHQase, aroK, aroC) in amino acid biosynthesis; genes for 3-oxoacyl-synthase II and fatty acyl-ACP thioesterase B in fatty acid biosynthesis (Table 1). In contrast, genes involved in degradation of these compounds were generally up-regulated, such as genes for α-amylase, NADP-specific glutamate dehydrogenase and enoyl-CoA hydratase/3-hydroxyacyl-CoA dehydrogenase (Table 1).

### Genes involved in phytohormone metabolism and signalling

Auxin-activated signalling pathway was enriched in the down-regulated DEGs (Fig. 3). A *YUCCA* gene encoding a flavin monooxygenase that converts indole-3-pyruvic acid (IPA) to indole-3-acetic acid (IAA) [24], was down regulated (Table 2). Other down-regulated auxin-related genes included an auxin efflux carrier *PIN3* gene together with seven *PIN-like* genes (Table S3), two auxin influx carrier *AUX/ LAX* genes, two phytochrome-interacting factor 4 (*PIF4*) genes and an auxin response factor *ARF* gene (Table 2). By contrast, genes participating in auxin catabolism and homeostasis, including genes for 2-oxoglutarate-dependent dioxygenase DAO and indole-3-acetic acid-amido synthetase GH3.6 were up regulated (Table 2). The DEGs included groups of early auxin responsive genes, *Aux/IAA* (8 DEGs up, 6 DEGs down), Grechen Hagen *GH3* (3 out of 4 down) and small auxin upregulated RNA (SAUR) genes (*SAUR50*, 1 DEG, up; *SAUR21*, 2 DEGs, down; *SAUR36*, 2 DEGs, down; Table S3).

**Table 2:** Genes involved in phytohormone metabolism and signalling

Description	Pattern of DEGs	Gene ID
***Auxin***		
Indole-3-pyruvate monooxygenase; YUCCA	down	oases_465475
2-oxoglutarate-dependent dioxygenase DAO	up	oases_546679, oases_546677, trinity_352891
Indole-3-acetic acid-amido synthetase GH3.6	up	oases_585901; rnaspades_20166
Auxin efflux carrier component 3; PIN3	down	oases_96998
Phytochrome-interacting factor 4; PIF4	down	oases_383788, oases_383793
Auxin influx carrier; AUX1 / LAX	down	trinity_70441, oases_184908
Auxin response factor; ARF	down	trinity_295296
***Gibberellin***		
Gibberellin 20 oxidase; GA20ox	down	oases_103383, oases_103382, oases_532982
Ent-kaurene oxidase; GA3	down	oases_118699, oases_118698
DELLA protein	up	trinity_376415, trinity_376421
***Brassinosteroids***		
Brassinosteroid-6-oxidase 1	down	oases_222582, trinity_237646, oases_222575
CYP90A1	down	oases_115434
***Strigolactones***		
All-trans/9-cis-β-carotene isomerase; DWARF27	down	oases_375815, oases_375817, oases_375816,
		rnaspades_110041, oases_375808
Strigolactone esterase; DWARF14	up	oases_468066
Strigolactone esterase; DWARF14	down	oases_448880, oases_448881
***Cytokinin***		
Adenylate isopentenyltransferase 5; IPT5	up	oases_535017, oases_535016; oases_535015
Histidine-containing phosphotransfer protein	up	oases_162595, oases_162591
Type-B ARABIDOPSIS RESPONSE REGULATOR	up	oases_337156
Type-A ARABIDOPSIS RESPONSE REGULATOR	down	oases_455999
WUSCHEL-related homeobox 1; WUS	up	oases_589420
***Abscisic acid***		
Zeaxanthin epoxidase; ZEP	down	oases_145318
SNF1-related protein kinase 2; SnRK2	down	trinity_216465, oases_147911
ABA responsive element binding factor; ABF	down	oases_140827
***Ethylene***		
1-aminocyclopropane-1-carboxylate oxidase	up	oases_571321, oases_522201, rnaspades_28915
Ethylene-responsive transcription factor ERF2	up	rnaspades_123541
***Jasmonic acid***		
Jasmonic acid-amino synthetase; JAR1_4_6	up	oases_262217, trinity_253178, oases_262230,
		oases_262216, oases_262227
Jasmonate ZIM domain protein; JAZ	down	rnaspades_58714, oases_151042, trinity_148341
***Salicylic acid***		
Isochorismate synthase; PHYLLO	up	rnaspades_8947, oases_243078
Salicylic acid 3-hydroxylase; DMR6	up	oases_327994
Pathogenesis-related protein 1; PR-1	up	rnaspades_100763, rnaspades_107927

Genes encoding gibberellin 20 oxidases (GA20oxs, 3 DEGs) converting GA_12_ or GA_53_ to GA_9_ or GA_20_, and genes encoding ent-kaurene oxidases (GA3s, 2 DEGs) catalyzing the oxidation of ent-kaurene to ent-kaurenoic acid [25], were down regulated in scion stems on marang rootstocks (Table 2). In contrast, genes encoding DELLA proteins (2 DEGs), and GID1b proteins (6 DEGs, Table S3), the GA receptors that interact with DELLA proteins [26], were upregulated.

Three isopentenyltransferase genes (*IPTs*) encoding the rate-limiting enzymes in cytokinin (CK) biosynthesis [27], together with genes encoding histidine-containing phosphotransfer proteins and the type B ARABIDOPSIS RESPONSE REGULATOR (ARR), were up-regulated in stems on dwarfing rootstocks (Table 2). A gene encoding transcription factor WUSCHEL, the downstream target of type-B ARRs [28], was highly upregulated (Fig. 2c).

Brassinosteroids (BRs) are growth-promoting steroid hormones regulating multiple aspects of physiological processes, and disruption in BR signalling is often characterized by profound morphological defect such as dwarfism [29]. Stem tissues on dwarfing rootstocks exhibited down-regulation in four genes participating in BR biosynthesis, including three brassinosteroid-6-oxidase genes and one *CYP90A1* gene (Table 2). However, expression of most genes related to BR transduction was not significantly changed. Strigolactones are a group of carotenoid-derived hormones that control shoot branching and diverse aspects of plant growth [30]. The strigolactone biosynthetic genes, *DWARF27* (5 DEGs) were highly down-regulated (log_2_FC ranged from -7.8 to -4.5) in stems on marang rootstocks (Fig. 2c; Table S3). The *DWARF14* genes that encode the α/β-hydrolase protein receptors involved in both strigolactone perception and deactivation Seto et al. [31], were differentially regulated in these tissues (Table 2).

Of the stress-related hormones, genes related to key components in ethylene transduction were up regulated in stems on dwarfing rootstocks (Table 2, Table S3). These included the upregulation of 3 DEGs encoding 1-aminocyclopropane-1-carboxylate oxidases (ACOs) and 20 DEGs encoding ethylene-responsive transcription factors, such as ERFs (6 DEGs), AP2/ERF (11 DEGs) and RAP2-3 (3 DEGs). In jasmonic acid (JA) signalling, genes encoding jasmonic acid-amino synthetases (JAR1, 5 DEGs) participating in the production of bioactive jasmonyl-L-isoleucine (JA-Ile), were up-regulated, while genes encoding jasmonate ZIM domain-containing proteins (JAZ, 3 DEGs), the negative regulators of JA signalling [26], were down-regulated in these tissues (Table 2). In salicylic acid (SA) signalling, two genes encoding isochorismate synthases, and a gene encoding salicylic acid 3-hydroxylase, an enzyme involved in biosynthesis of 2,5-dihydroxybenzoic acid [32], together with two *PR-1* genes, were up-regulated in scions stems on dwarfing rootstocks (Table 2).

A down-regulated gene encoding zeaxanthin epoxidase (ZEP) was the only ABA biosynthetic gene detected in DEGs (Table 2). However, a total of 10 DEGs encoding abscisic acid 8'-hydroxylases, the enzyme involved in ABA catabolism [33], were down-regulated (Table S1). A gene encoding ABA responsive element binding factor (ABF) was down-regulated, along with genes encoding the basic leucine zippers (b-ZIP, 8 out of 11 DEGs, Table S4).

### Genes involved in cell wall biosynthesis and cell expansion

Genes encoding cellulose synthase CesA1 (4 DEGs), CesA2 (3 DEGs), CesA3 (2 DEGs) and CesA6 (1 DEG) were down regulated in scions on marang rootstocks (Table 3). The down-regulated DEGs included three genes encoding xyloglucan galactosyltransferases (XLT2 or MUR3) participating in xyloglucan biosynthesis and two genes encoding xyloglucan endotransglucosylase/hydrolases (XTH6), the wall re-modellling enzymes [34]. There were 5 DEGs encoding galactan beta-1,4-galactosyltransferases, the pectin biosynthetic enzymes, and 11 DEGs encoding pectin acetylesterases, the enzymes involved in pectin deacetylation, all were down-regulated in these tissues (Table S3). The mannan endo-1,4-beta-mannosidase genes (2 DEGs) and the pectate lyase genes (17 DEGs) were also down-regulated (Table 3, Table S3). However, two DEGs encoding glucan endo-1,3-beta-glucosidases, the enzymes involved in degrading callose (ß-1,3 glulcan), were up-regulated.

**Table 3:** Genes involved in cell wall biosynthesis and cell expansion

Description	Expression pattern	Gene ID
Cellulose synthase CesA1	down	trinity_378765, trinity_255101,
		rnaspades_58319, trinity_255104
Cellulose synthase CesA3	down	oases_96831, oases_96828
Cellulose synthase CesA2	down	oases_240822, oases_240814, oases_240836
Cellulose synthase CesA6	down	oases_240813
Xyloglucan endotransglucosylase/hydrolase, XTH6	down	oases_212485; trinity_276618
Xyloglucan galactosyltransferase; XLT2/MUR3	down	oases_502036, oases_721460; trinity_128650
Galactan beta-1,4-galactosyltransferase	down	oases_413218, oases_413207,
		oases_413211, oases_413210, oases_413215
Mannan endo-1,4-beta-mannosidase	down	oases_287541, oases_287540
Glucan endo-1,3-beta-glucosidase	up	oases_477865, oases_477864, oases_324390
Pollen-specific LRR extensin-like protein	down	oases_125652
Wall-associated receptor kinase; WAK	down	trinity_59924, oases_261741, oases_203194
		oases_261744, oases_261743
Fasciclin-like arabinogalactan protein; FLA	down	oases_218581, oases_218579,
		trinity_28403, trinity_173278

Expansin is a family of cell wall proteins that mediate the acid-induced cell expansion through their non-enzymatic cell wall loosening ability [34]. There were 29 DEGs identified as expansins, all were down-regulated in scion stems on marang rootstocks (Table S3). Five DEGs encoding the wall-associated receptor kinases (WAKs), a family of pectin receptors required for cell expansion [35], and 4 DEGs encoding the fasciclin-like arabinogalactan proteins (FLAs), were down-regulated in the same tissues (Table 3). Genes involved in cell separation and cell wall modification [34], such as the polygalacturonase (20 out of 21 DEGs) and pectinesterase genes (8 DEGs) were also down-regulated (Tale S2).

### Genes involved in secondary metabolism

**Fig. 5:**
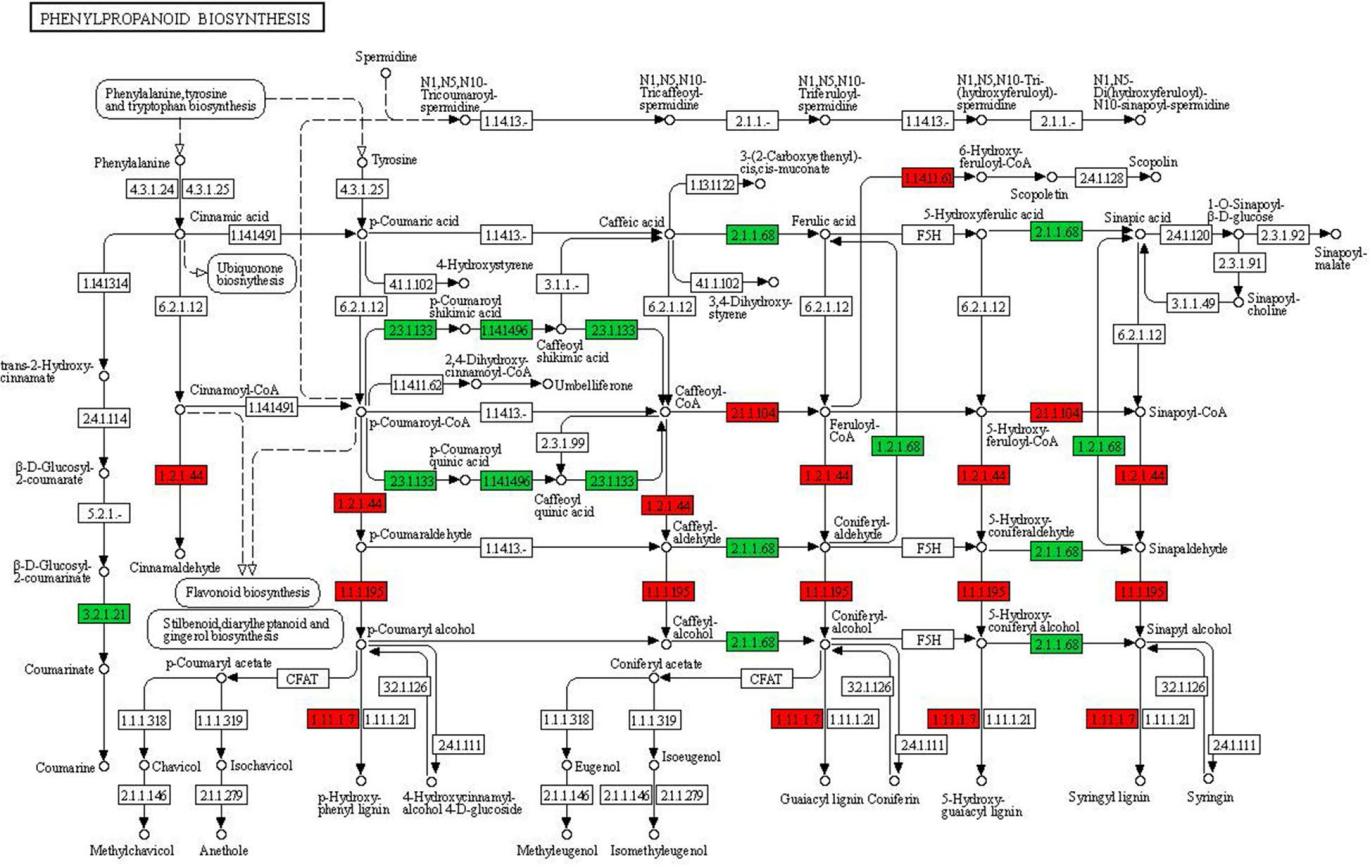
Differentially expressed genes in phenylpropanoid biosynthesis pathways. RNA-sequencing data were analysed by KEGG. Up-regulated genes, red; down-regulated genes, green; [EC:1.14.11.61] feruloyl-CoA 6-hydroxylase; [EC:1.2.1.44] cinnamoyl-CoA reductase; [EC:1.1.1.195] cinnamyl-alcohol dehydrogenase; [EC:2.1.1.104] caffeoyl-CoA O-methyltransferase; [EC:3.2.1.21] beta-glucosidase; [EC:1.2.1.68] coniferyl-aldehyde dehydrogenase; [EC:2.3.1.133] shikimate O-hydroxycinnamoyltransferase; [EC:2.1.1.68] caffeic acid 3-O-methyltransferase; [EC:1.14.14.96] p-coumaroyl ester 3'-hydroxylase

The phenylpropanoid pathway provides precursors for numerous secondary metabolites, including monolignols and flavonoids [36]. KEGG analysis indicated enrichment of the pathway in DEGs (Fig. 3). Specially, genes encoding p‐coumaroyl ester 3‐hydroxylases (C3′H, 3 DEGs) and quinate/shikimate p-hydroxycinnamoyltransferases (HCT, 5 DEGs) were down regulated in stems on marang rootstocks, while genes involved in the latter steps of the phenylpropanoid pathway were upregulated (Table 4, Fig. 5). The activity of both C3’H and HCT provides entry into the main lignin biosynthetic pathway leading to the formation of G‐ and S‐type monolignols [37]. Flavonoids are crucial for plant growth and stress tolerance. A total of 12 DEGs involved in flavonoid biosynthesis were upregulated (Table 4) in stems on dwarfing rootstocks. These included a chalcone synthase (CHS) gene involved in the first committed step in flavonoid biosynthesis [38], a bifunctional dihydroflavonol 4-reductase/flavanone 4-reductase gene participating in the late key step for the biosynthesis of anthocyanins and proanthocyanidins [38], a flavanone 3-hydroxylase (F3H) gene and an anthocyanidin synthase gene.

**Table 4:** Genes involved in secondary metabolism

Description	Expression pattern	Gene ID
***Phenylpropanoid biosynthesis***		
Caffeic acid 3-O-methyltransferase	down	trinity_267412, oases_384295
Coniferyl-aldehyde dehydrogenase	down	trinity_320214, oases_445900, trinity_320212
Shikimate O-hydroxycinnamoyltransferase; HCT	down	oases_375831; trinity_347573,
		oases_375847, trinity_44469, oases_375829
P-coumaroyl ester 3'-hydroxylase; C3'H	down	oases_4947, oases_4919, oases_4943
ß-glucosidase	down	oases_241103, oases_241107, oases_278543
Caffeoyl-CoA O-methyltransferase	up	oases_399468
Cinnamyl-alcohol dehydrogenase; CAD	up	trinity_96535, oases_222834
Cinnamoyl-CoA reductase; CCR	up	oases_222835, oases_222840, oases_222837
Feruloyl-CoA 6-hydroxylase; F6H	up	oases_511410
***Flavonoid biosynthesis***		
Bifunctional dihydroflavonol 4-reductase/ flavanone 4-reductase	up	trinity_95243
Chalcone synthase; CHS	up	trinity_18046
Flavanone 2-hydroxylase	up	oases_557081
Flavanone 3-hydroxylase	up	oases_327990
Isoflavone 2'-hydroxylase	up	trinity_142768
Isoflavone 3'-hydroxylase	up	trinity_142768, oases_517633, oases_531828
Anthocyanidin synthase	up	oases_532538
Anthocyanidin 3-O-glucosyltransferase	up	trinity_193322
Polyphenol oxidase	up	oases_488858, oases_488859
***Isoprenoid biosynthesis***		
1-deoxy-D-xylulose-5-phosphate synthase; dxs	down	oases_47553, oases_47552, oases_128123,
		oases_128124, oases_475380, oases_128110
Hydroxymethylglutaryl-CoA reductase; HMGR	down	trinity_239528, oases_223526, oases_223530
Farnesyl diphosphate synthase	down	oases_327788
Geranylgeranyl diphosphate synthase	down	trinity_12151
Squalene synthase	down	oases_109614, oases_109610
Myrcene synthase	down	oases_421237
Pinene synthase	down	oases_372831

Genes involved in isoprenoid biosynthesis were generally down-regulated in scion stems on marang rootstocks, such as genes encoding myrcene synthase, pinene synthase, squalene synthase and linalool synthase, together with 6 DEGs encoding ß-caryophyllene synthases / sesquiterpene synthases (Table 4, Table S3).

### Genes involved in redox homeostasis and defense response

Large numbers of ROS-related genes were upregulated in scion stems on dwarfing rootstocks (Table 5), such as the peroxidase (4 DEGs, one with logFC > 7), glutathione transferase GST23 (2 DEGs) and thioredoxin 1 (4 DEGs) genes. The upregulated DEGs also included a gene encoding a heme oxygenase (HO-1) involved in heme cleavage and production of biliverdin, a strong antioxidant cytoprotectant [39], and 2 genes encoding peroxygenases, a group of hydroperoxide-dependent oxygeneases involved in oxylipin production [40]. Key genes for biosynthesis of thiamine, a potent antioxidant, were also up-regulated in these tissues (Table 5, Table S3), including genes encoding thiamine thiazole synthases (THI1, 3 DEGs), cysteine-dependent adenosine diphosphate thiazole synthase (THi4, 1 DEG) and phosphomethylpyrimidine synthases (thiC, 8 DEGs).

**Table 5:** Genes involved in redox homeostasis and defense response

Description	Expression pattern	Gene ID
Peroxidase	up	oases_483367, rnaspades_62572,
		trinity_416258, oases_558385
Thioredoxin 1; trxA	up	trinity_210271, oases_556771,
		oases_247714, trinity_70776
Monothiol glutaredoxin-S2	up	oases_687633, oases_583211
NADH-cytochrome b5 reductase	up	oases_578662
Glutathione transferase GST23	up	oases_550971, oases_550970
Heme oxygenase	up	oases_455683
Calcium-binding peroxygenase; PXG	up	oases_563256, oases_563253
Calmodulin-like protein CML29	up	oases_534040
Cyclic nucleotide gated channel; CNGC	up	oases_362079, oases_362096, trinity_18066
Calcineurin B-like	up	oases_147942; oases_147949
Molecular chaperone; sHSP	up	oases_91766, oases_442070
Thiamine thiazole synthase; THI1	up	rnaspades_29403, oases_282730, trinity_212353
ADP-thiazole synthase; THi4	up	trinity_212354
Voltage-dependent anion channel protein 2; VDAC2	up	oases_237411, oases_237421
Serine/threonine-protein phosphatase 2B regulatory	up	oases_152721, rnaspades_72610

Genes encoding key components in the MAPK cascade, calcium signalling and the NOD-like receptor signalling pathway were upregulated in stems on dwarfing rootstocks (Table S4, Table S3). These included genes encoding the calmodulin-like protein CML29, the calcineurin B-like proteins, the cyclic nucleotide gated channels participating in stress-induced Ca_2_^+^ influx [41], the voltage-dependent anion channels, the serine/threonine-protein phosphatase 2B regulatory subunit (Table 5), along with large numbers of WRKY transcription factors (16 DEGs), the cathepsins family members (14 DEGs) and the basic endochitinase / chitinase family members (6 DEGs, Table S3). A total of 25 out of 28 DEGs identified as disease resistance (R) genes were upregulated in these tissues, including R gene candidate *RGA* (oases_423898 and trinity_250424), *RPS2* (rnaspades_2531, rnaspades_118057), *RPS5* (rnaspades_11681) and *RPM1* (oases_397796, oases_397788 and oases_261017), and a total of 30 genes encoding subtilisin-like protease family members were deferentially expressed (Table S3).

### Validation of DEGs using qRT-PCR

**Fig. 6:**
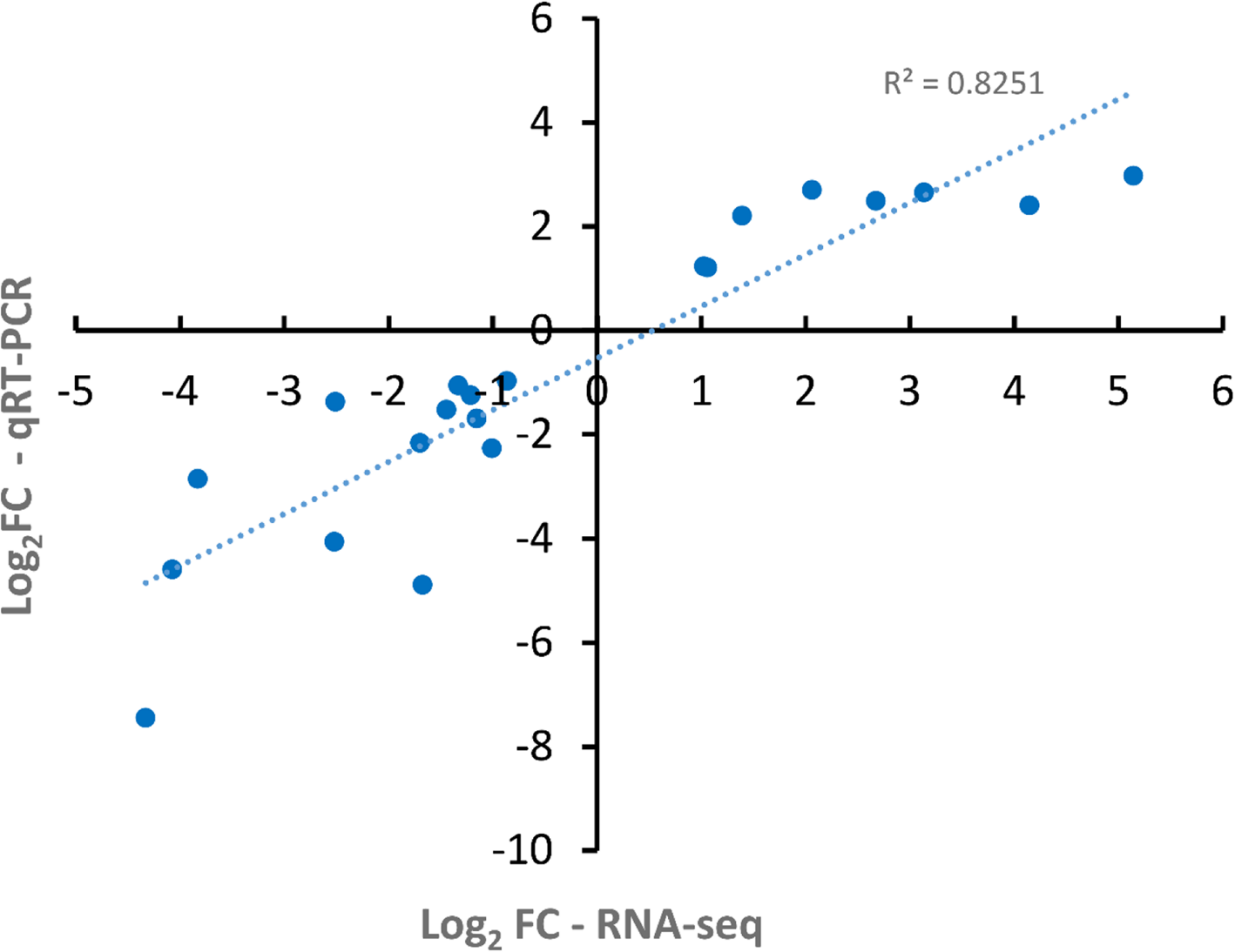
Correlation of gene expression pattern between RNA-Sequencing and qRT-PCR

A random selection of 21 DEGs analysed above was validated by qRT-PCR. These relative expression levels were presented as log_2_FC (fold change of marang rootstocks vs. self-graft), and plotted against the log_2_FC value of each gene detected by the RNA-seq method (Fig. 6). The results showed both qRT-PCR and RNA-seq displayed similar expression patters for the genes tested, with the correlation coefficient, *r* = 0.9083, *P* < 0.01, indicating a high correlation between both methods.

## Discussion

Breadfruit plants growing on marang rootstocks were characterised by significant inhibition in stem elongation growth leading to a dwarf phenotype with over 50% reduction in plant height (Table 1, also see [3]). Transcriptome analysis in scion stems showed these plants displayed down-regulation in key genes for biosynthesis of amino acids, lipids, and cell wall, reflecting a reduction in carbon utilisation for stem growth. In sink organs sucrose is hydrolysed by invertase and/or sucrose synthase in order to make hexose for energy production, metabolism and macromolecule biosynthesis [42]. The higher sucrose levels but lower hexose levels in stems on dwarfing rootstocks (Fig. 1) were consistent with the fact that these tissues exhibited significantly lower transcription and enzyme activity of both SAI and NIV (Fig. 4), reflecting a disruption in sucrose utilisation. The increase in the relative contribution of sucrose synthase to sucrose degradation as indicated by higher sucrose synthase activity (Fig. 4) may represent an adaptation strategy for energy conservation in these tissues, as conversion of sucrose to hexose phosphates via sucrose synthase uses only half the ATP needed for conversion via invertase [43]. However, the build-up of sucrose in these tissues suggests the gross increase in sucrose synthase activity is not able to compensate for the loss of hydrolysis. Furthermore, normal plant growth could be adversely impacted by the repression of *SAIs* that are associated with elongation growth [44, 45]. Reduction in SAI activity as a result of disruption in a *WAK* gene leads to loss of cell expansion [35]. Notably, four *WAK* genes were down regulated in scion stems on marang rootstocks (Table 3). Suppression of *NIVs* also leads to sever defects in cell elongation [43, 46].

High sucrose concentration has been shown to negatively regulate sucrose influx through repressing the transcription and activity of sucrose transporters [47–49]. Concurrently, a large array of genes encoding nutrient transporters/facilitators, including sugar transporters (SUT1, SUT4, SWEETs), amino acid transporters, choline transporters-like, K^+^ channels and aquaporins were down-regulated in scion stems on marang rootstocks (Table 1). Many nutrient transporters function as secondary active transporters coupled with proton electrochemical gradient generated by the activity of PM H^+^-ATPase. The down-regulation of these genes, together with the decreased activity of PM H^+^-ATPase (Fig. 4, also see [3]) could affect passage of nutrients (sucrose, hexose, amino acids) across cell membranes. Specifically, during the long-distance transport of sugars, SUTs or SWEETs together with K^+^ channel AKT2/3 facilitate sucrose retrieval to maintain the hydrostatic pressure along the phloem pathway [23, 50]. Sucrose unloading to stem tissues in woody species may also be impacted by *SUTs* that pay active role in sucrose release to adjacent phloem parenchyma cells and sucrose exchange between xylem and phloem cells [51, 52]. The subcellular compartmentation of sucrose could be impaired by the suppression of a gene encoding the tonoplast‐localized SUT4 that functions in retrieving sucrose from vacuole for downstream processing [53]. Furthermore, the symplastic transport could be impacted by the suppression of the choline transporter-like genes that are involved in plasmodesmata maturation and sieve plate development [54, 55]. The downregulation of these nutrient transporter genes, together with the decrease of invertase activity, point to the role of carbon partitioning in the development of dwarf phenotype on marang rootstocks.

PM H^+^-ATPase plays a central role in promoting cell expansion via the acid growth mechanism [56]. Scion stems on marang rootstocks displayed decreased PM ATPase activity accompanying with down-regulated genes for key players [34] involved in the acid growth mechanism. These included genes encoding invertases and K^+^ channel AKT1 that provide turgor pressure to drive cell expansion [35, 57], expansins, WAKs, and key enzymes for biosynthesis and re-modelling of cellulose, xyloglucans and pectins (Table 3, Table S3). It has been shown that transcript abundance of some *FLA*s is positively correlated with the onset of secondary-wall deposition and wood properties in xylem fibres of various tree species [58]. Noticeably, four *FLAs* were expressed at lower levels in stems on dwarfing rootstocks (Table 3). Consistent with the impact on secondary-cell wall, down-regulation of lignin biosynthetic genes, *HCT* and *C3’H*, was shown in these tissues (Table 4), suppression of *HCT* or *C3’H* limits the flux to coniferyl and sinapyl alcohols (Fig. 5), leading to decreased lignin content and stunted growth [37]. The down-regulation of lignin biosynthesis pathway in breadfruit scions on marang rootstocks is consistent with findings reported for scions of apple trees grafted on dwarfing rootstocks ‘M27’ and ‘M9’ [14].

Cell expansion could be inhibited by the down-regulation of the *SAUR21* genes that regulate the activity of PM H^+^-ATPase [59]. Auxin biosynthesis could be suppressed by the down-regulated *YUCCA* gene that regulates a rate-limiting step in IAA biosynthesis via IPA pathway [24]. Polar auxin transport was likely disrupted by the down-regulation of both auxin efflux and influx carrier genes (Table 2). The results are consistent with the role of auxin in rootstock-induced dwarfing of other species [9, 13]. GA biosynthesis in scion stems on marang rootstocks could be inhibited by the down-regulation of both *GA20oxs* and *GA3s*. The results agree with our previous findings showing increased abundance of DELLA proteins, repressors of GA signalling, in the tissues [4]. Both the transcription of auxin efflux carrier *PIN3* and the activity of PIFs could be inhibited by the over-accumulation of DELLA proteins [26, 60]. The up-regulation of the three CK biosynthetic genes, *IPTs* suggests the capacity of stem-localised synthesis of CK could be elevated to compensate for the under-supply of CK suggested from dwarfing rootstocks [9]. Transcription of *IPTs* could be mediated by the down-regulation of auxin signalling [27]. On the other hand, both auxin efflux and influx carriers are among the downstream targets of type-B ARRs [28]. The roles of strigolactones in breadfruit dwarfing could be suggested by the three strongly repressed *DWARF27* genes (Table S3). Plants with disrupted *DWARF27* display dwarf habit [30]. The down-regulation of genes encoding brassinosteroid-6-oxidases and CYP90A1 may suggest disruption in BR biosynthesis, as both proteins catalyse multiple oxidative conversions essential for BR biosynthesis, suppression at either brassinosteroid-6-oxidase or *CYP90A1* gene exhibits severe dwarf phenotype with defective cell elongation and very short internodes [29].

The upregulated genes from stems on marang rootstocks were enriched for ROS-related genes and signalling pathways of SA, JA and ethylene. The upregulation of MAPK cascade, calcium signalling and the nucleotide-binding leucine-rich repeat (NB-LRR) proteins encoding by *R* genes has been described as an innate immune response in plant similar to the NOD-like receptor signalling used in animal for amplification of defence signals [61, 62]. While these pathways are often involved in quick and robust responses to stress signals such as activation of defense-related genes, localized programmed cell death and stress adaptation, growth inhibition is often observed as a trade-off in plants with strong defense response [62]. Repression of auxin signalling has been demonstrated as part of the SA-mediated response [63]. The JAZ repressors in the JA signalling pathway interact with DELLA proteins to prioritize defense over growth [26]. The NOD-like innate immune response was also shown to suppress growth through DELLA stabilization [64]. Suppression of lignin biosynthetic gene *HCT* was shown to trigger SA signalling pathway [65]. On the other hand, repression of lignin biosynthesis has been linked to the redirection of metabolic flux toward flavonoids [66, 67]. Flavonoids are natural inhibitors of auxin transport [68]. Sucrose also induces the accumulation of anthocyanin (a kind of flavonoids) [69, 70], and this induction was found to be positively mediated by DELLA proteins which was also stabilized by sucrose [71]. Sucrose acts on carbon partitioning and regulation of phloem transport [47, 49], also interacts with various hormones such as GA, JA and SA signalling [72]. Therefore, disruption in the efficient invertase-mediated conversion of sucrose to glucose and fructose, not only limits nutrient and energy supply, but also disrupts the complex hormone interaction and the crosstalk between growth and defence.

## Conclusion

Combined RNA-seq transcriptomic profiles and biochemical analysis suggest breadfruit plants growing on marang rootstocks exhibit disruption in nutrient transport and sucrose utilization for stem elongation growth. Decrease in the activity of PM-H + -ATPase, in combination with the down-regulation of genes encoding various cell wall proteins and enzymes involved in turgor regulation, biosynthesis/re-modelling of cellulose, xyloglucans, pectins, and lignin is proposed to impair cell expansion and cell wall biosynthesis of both primary and secondary cells, leading to a dwarf phenotype. Auxin and GA signalling pathways, together with brassinosteroid and strigolactone biosynthetic genes dominated the differentially down-regulated genes, suggesting disruption of these transduction networks play a role in the rootstock-included dwarfing. The large numbers of upregulated genes involved in defence response, including stress-related hormone signalling pathways, the MAPK cascade and the NOD-like receptor signalling, are proposed to suppress plant growth, and contribute to the development of dwarf phenotype.

Rootstock-induced dwarfing is a complex mechanism. Our results support that networks of multiple pathways integrated with carbon partitioning disrupt the balance of growth and defence leading to inhibition of cell elongation growth. Assessment for the long-term effect of marang rootstocks on breadfruit phenotype is required in the future. The valuable gene resource generated in the current work can help facilitate the functional study and continuous characterisation of the underlying molecular processes involved in the interspecific scion/rootstock interaction that leads to breadfruit dwarfing. Furthermore, the information provides opportunity to design screening strategies to assist in breeding and selection of potential size-controlling rootstocks. Although in orchard practice, breadfruit tree height may be controlled though the use of plant growth retardants, such as paclobutrazol [73], for woody species, this generally involves a long-term, repeated application of these chemicals in order to achieve effective tree-size reduction. Development and characterisation of dwarfing rootstocks provides a sustainable solution to breadfruit tree vigor control.

## Methods

### Plant materials

Breadfruit (*Artocarpus altilis* cv. Noli) and marang (*Artocarpus odoratissimus*) plants were obtained from a commercial nursery at Cairns, northern Queensland. Breadfruit plants, as rooted cuttings, and marang plants as seedlings, were grown under glasshouse condition at 25–28 °C with natural daylight and daily water supply. Plants were grown in pots containing vermiculite and soil mixture as described previously [3]. Breadfruit scions selected from plants of 30 ~ 50 cm tall were grafted onto marang seedlings of similar sizes through approach grafting [74]. As a control, breadfruit scions were also grafted onto breadfruit rootstocks of the same cultivar (self-graft). Each established grafted plant was transferred to an 85-Litre pot six months after grafting, and continued to grow under the same condition. For growth comparison, the own-rooted breadfruit plants (non-graft) were also grown alongside under the same condition.

Growth parameters including elongation rate of scion main stems and branches, final scion height, scion stem diameter, node number, branch number, internode length, leaf size and leaf number, were examined at 26 months after grafting as previously described [3]. Ratio of stem diameter above and below graft union was calculated from the stem circumference measured at 5 cm above and below the graft union. Leaf characters, including leaf colour and vein colour, shape of leaf base, apex and margin, and lobe number were also examined at the same time. Three fully expanded leaves (source leaves) of each biological replicate were selected for measurement of chlorophyll concentration. Three circular disks were punched from each leaf and immediately extracted with 96% ethanol. Concentration of chlorophyll *a* and *b* was calculated from the absorbance at 648 nm and 664 nm according to the method of Lichtenthaler [9]. Assessment of graft compatibility was carried out every 12 months in the period from 3 to 26 months after grafting. Results from two independent trials (each with 8 replicates for each graft combination from the start) were recorded as 1-year and 2-year graft compatibility rates out of the survivals at 3 months after grafting. Collected data from different rootstocks were tested with Fisher’s exact test. Only compatible grafts were used for down-stream analysis, including growth comparison, biochemical and gene expression analysis. Plants displaying graft compatibility were defined as showing active growth with progressive development of new internodes from apical buds or axillary buds, and continuous emergence of green leaves from the apex (Figure S1). Visual inspection of graft union revealed no fissure across all grafts. Anatomy examination of graft interface was further carried out on three interspecific grafts at 26 months, after their internode and leaf samples were collected for downstream assay. To achieve this, graft unions were sawed to reveal both the cross-sections and longitudinal sections. Graft incompatibility was visually evaluated using a five-category rating (from A to E) according to Mosse and Herrero [22]. Category A represented a perfect union with the graft line between bark and wood hardly visible. Category B indicated good unions, with continuous bark and wood, although the line of union in the wood was often distinguished by excessive ray formation. Category C was characterized by bark discontinuities and category D by vascular wood discontinuities. Category E involved breakage of graft unions [22].

### Non-structural carbohydrate analysis

Upon removal from plants, stem tissues at the second internode sections were snap-frozen in liquid N_2_. Thereafter, soluble sugars of each stem sample (50 mg) were triple-extracted in 80% ethanol at 60 °C for 2 h. Supernatants were evaporated and dissolved in sterile water. Levels of soluble sugars were determined enzymatically, with glucose and fructose according to D-Fructose/D-Glucose Assay Kit (Megazyme, Bray, Ireland), and sucrose according to Birnberg and Brenner [75]. The pellets of ethanol extraction were resuspended in 0.1 N NaOH and boiled for 30 min. After adjusting the pH to 5, starch was digested overnight at 50 °C with 1000 U of amyloglucosidase and 20 U of α-amylase, and determined enzymatically for glucose units according to a starch-determination kit (Megazyme, Bray, Ireland).

### Enzyme assay

For the extraction of sucrose-metabolizing enzymes, fresh stem tissues (0.5 g) of the second internodes counted from the top were immediately frozen and ground to powder under liquid N_2_, homogenized with 1.5 mL extraction buffer following the procedure of Mirajkar et al. [76]. The activity of invertase was measured at 37 °C by adding 50 µL of dialysed enzyme to an assay mix containing 90 mM sodium acetate buffer pH 4.5 (acid invertase) or pH 7.5 (neutral invertase), and 60 mM sucrose as previously described [76]. Glucose assay was then performed as above. One unit of invertase activity was defined as the amount of enzyme that formed 1 µmol of glucose in 1 min under the assay condition. The activity of sucrose synthase was measured according to Tomlinson et al. [77]. Fructose assay was then performed as above. Sucrose synthase activity was expressed in µmol fructose min^−1^ mg^−1^ Protein. For extraction of plasma membrane H^+^-ATPase, microsomal fraction was prepared from the same stem tissues following a previous protocol [74]. ATP hydrolytic activity was measured at 38 °C with microsomal suspension added into a reaction mix [50 mM Tris-MES, pH 6.5 with 3 mM Tris-ATP, 50 mM KCl, 3 mM MgSO_4_, 0.5 mM ammonium molybdate, 50 mM KNO_3_ and 0.1% (w/w) Triton X-100], followed by inorganic phosphate assay as previously described [74]. The activity of vanadate-sensitive ATPase was calculated from the difference between activities in the absence and presence of 1 mM Na_3_VO_4_. Protein concentration was determined according to Bradford [78].

### RNA isolation, cDNA-library construction and Illumina sequencing

RNA extraction for transcriptomic profiling was performed from the main stems of breadfruit scions at 22 months after grafting. A stem section at the second internode of each grafted plant was sampled as one biological replicate. Three replicates were performed for each graft combination. Upon removal from plants, stem tissues were immediately immersed in RNA*later* (Life Technologies, Australia), before stored at -80 °C. Total RNA was extracted by using RNeasy kit with on-column DNase digestion (Qiagen, Australia). Since there was no reference genome for breadfruit species, a reference library was prepared at the same time to assist de novo assembly. To achieve this, a mixed RNA library was generated with equal combination of total RNA of stem sections at the apex, and of every internode from the top to the sixth internode of each replicate. Total RNA quality was verified by the integrity of the 25S and 18S RNA with an Agilent 2100 Bioanalyzer (Agilent, USA). cDNA libraries were prepared using a TruSeq® Stranded Total RNA Library preparation kit with Ribo-Zero Plant (Illumina, USA) and indexed with unique nucleic acid identifiers (Illumina TruSeq V2 index sequence). All the indexed cDNA libraries were pooled to generate one multiplexed sample for one lane of a SP flow cell (Illumina, USA). An unequal sample pooling was performed so that the reference library took half of the lane, and the six libraries (three from marang rootstocks and three from the self-graft) evenly shared another half of the lane. The final mixed cDNA pool was sequenced using 150 bp paired-end sequencing strategy on an Illumina NovaSeq 6000 platform (Ramaciotti Centre for Genomics, University of New South Wales, Sydney).

### De novo assembly and annotation of transcriptome library

Filtered reads from cDNA libraries were pooled and assembled de novo into contigs by Australian Genome Research Facility (AGRF, Brisbane) following the protocol described by Cerveau and Jackson [79]. Briefly, three independent assemblies were performed with Trinity (k-mer = 55 [80]), Oases (k-mer = 53, 63, 73 [81]) and rnaSPAdes (k-mer = 55, 77 [82]). Results from each assembly were clustered with CD-HIT-EST with parameters: -G 0 –c 1.00 –aS 1.00 –aL 0.005 [83] and representative transcripts from each assembly were pooled. Transdecoder [84] was used to extract open reading frames (ORF) longer than 100 amino acids. The coding sequences were further clustered using CD-HIT-EST with parameters: -c 0.98 -G 0 -M 16,000 -T 16 -aS 1.0 -aL 0.05 [83]. Representative coding sequences were used for annotation in downstream analysis and the contigs where they originated were used as the final transcriptome assembly. Benchmarking Universal Single-Copy Orthologs (BUSCO) was used to identify universal single copy orthologs and to estimate the completeness of the assembly. Reads were mapped back to the assembly to assess mapping efficiency.

Peptide sequences obtained from the final transcriptome assembly were used for functional annotation using software InterProScan v5.28–67.0 (https://www.ebi.ac.uk/interpro/). KEGG annotation was carried out in KAAS (https://www.genome.jp/tools/kaas/). Additionally, assembled unigene sequences were annotated by alignment to the public databases: NCBI nr (non-redundant, see http://www.ncbi.nlm.nih); Swissprot (http://www.uniprot.org/), and COG (https://www.ncbi.nlm.nih.gov/COG/) with a threshold of e ≤ 10^–5^. A BLAST search was also performed against the genome of *Morus indica* L. (Moraceae), the most related species with a well-annotated and publicly available genome sequence in NCBI database.

### Differential gene expression and pathway analysis

EdgeR package v. 3.16.5 (https://bioconductor.org/packages/release/bioc/html/edgeR.html) was used to perform differential expression analysis. Only genes displaying at least 1 cpm (count per million reads) in any 3 samples were retained for further analysis. A generalized linear model (GLM) was used to perform differential expression comparison between the groups. Genes were considered differentially expressed if their FDR < 0.05 and |log_2_ fold change|≥ 1. R package topGO v2.36.0 (https://bioconductor.org/packages/release/bioc/html/topGO.html) was used to perform pathway analysis on differentially expressed genes. Analysis of Gene Ontology (GO) and Kyoto Encyclopedia of Genes and Genomes (KEGG) enrichment was tested by Fisher’s exact test.

### Quantitative real-time PCR

Aliquots of RNA samples were converted to cDNA with SuperScript reverse transcriptase and oligo(dT) (Life Technologies, Australia). Real-time PCR was performed on a Corbett Research Rotor-Gene 6000 cycler with the QuantiFast SYBR Green PCR Kit (Qiagen, Australia) as previously described [4]. Thermocycling was initiated with a 5-min incubation at 95 °C, followed by 45 cycles (95 °C for 10 s; 60 °C for 30 s). The specificity of amplification was confirmed by high-resolution melt curve analysis at the end of each run. The efficiency of each primer set (see Table S4 for primer sequences) was evaluated by standard curves using serial dilutions of cDNA samples. Each reaction was carried out in duplicate (technical repeat) with non-reverse-transcribed cDNA (RT^–^) as negative controls (non-template control). Four housekeeping gene candidates, elongation factor 1-*a*, actin, α-tubulin and γ-tubulin were tested for stability in scion stem tissue as previously described [4]. The expression of each gene was an average of three biological replicates.

### Statistical analyses

Statistical analysis of all data was performed in SPSS (IBM Statistics version 24). Significant differences related to growth rate, stem height and diameter, metabolite concentration, enzyme activity and gene expression levels were tested using analysis of variance (ANOVA) followed by Tukey's multiple comparison test at *P* < 0.05. Significant differences in number of nodes, branches and leaves were tested with Kruskal–Wallis test. Pathway enrichment and significant differences of 1-year and 2-year compatibility rates were tested by Fisher’s exact test.

## Supplementary Information

**Additional file 1: Table S1**. Morphological assessment of breadfruit plants growing on different rootstocks*. The 1-year and 2-year compatibility rates represent mean ± SD of two independent trials, each with eight biological replicates in each graft combination from the start. All other values represent mean ± SE of six biological replicates. Values with different letters from the same row are significantly different (*p* < 0.05). * Final measurement at 26 months after grafting.

**Additional file 2: Table S2**. *de novo* transcriptome assembly metrics.

**Additional file 3: Table S3**. Gene annotation and expression levels showing all differentially expressed genes (FDR < 0.05 and |logFC| ≥1)*. *LogFC, Log_2_-fold change of expression from samples on marang rootstocks vs those on self-graft; FDR, adjusted *p*-value for multiple hypothesis testing; logCPM, average log count for the gene across all samples; F, quasi-likelihood F-statistic for genes across all samples; C1-C3, raw gene counts for replicates of self-graft control; T1-T3, raw gene counts for replicates on marang rootstocks.

**Additional file 4: Table S4**. Quantitative real-time PCR primers.

**Additional file 5: Figure S1**. Representatives of graft-compatible phenotype of breadfruit plants growing on marang rootstocks (a) and self-graft (b) in the period from 3 months to 26 months after grafting.

**Additional file 6: Figure S2**. Representative images of longitudinal and cross sections through a graft union of breadfruit scion grafted on marang rootstock. a) Graft union from approach graft; b) Longitudinal section of a graft union; c) Cross-section of a graft union. Graft lines are indicated by arrows.

## Data Availability

The raw reads of RNA-sequencing and the de novo assembly of the reference transcriptome library supporting this article are available in NCBI BioProject database (https://submit.ncbi.nlm.nih.gov/subs/bioproject/SUB8554792/overview) with accession number: PRJNA679862. Lists of differentially expressed genes related to scion stems are provided in Supplementary Table S3.

## References

[1] Jones AMP, Ragone D, Tavana NG, Bernotas DW, Murch SJ (2011). Beyond the bounty: breadfruit (*Artocarpus altilis*) for food security and novel foods in the 21st century. Ethnobot Res Appl.

[2] Zhou Y, Taylor MB, Underhill SJR (2014). Dwarfing of breadfruit (*Artocarpus altilis*) trees: opportunities and challenges. Am J Exp Agric.

[3] Zhou Y, Underhill SJR (2019). A dwarf phenotype identified in breadfruit (Artocarpus altilis) plants growing on marang (A. odoratissimus) rootstocks. Horticulturae.

[4] Zhou Y, Underhill SJR (2020). Expression of gibberellin metabolism genes and signalling components in dwarf phenotype of breadfruit (*Artocarpus altilis*) plants growing on marang (*Artocarpus odoratissimus*) rootstocks. Plants (Basel).

[5] Soumelidou K, Battey NH, John P, Barnett JR (1994). The anatomy of the developing bud union and its relationship to dwarfing in apple. Ann Bot.

[6] Basile B, Marsal J, Solar LI, Tyree MT, Bryla DR, Dejong TM (2003). Hydraulic conductance of peach trees grafted on rootstocks with differing size-controlling potentials. J Hortic Sci Biotech.

[7] Atkinson CJ, Else MA, Taylor L, Dover CJ (2003). Root and stem hydraulic conductivity as determinants of growth potential in grafted trees of apple (Malus pumila Mill.). J Exp Biol.

[8] Olmstead MA, Lang NS, Lang GA (2010). Carbohydrate profiles in the graft union of young sweet cherry trees grown on dwarfing and vigorous rootstocks. Sci Hortic.

[9] Lochard RG, Schneider GW (1981). Stock and scion growth relationships and the dwarfing mechanism in apple. Hortic Rev.

[10] Tworkoski T, Fazio G (2016). Hormone and growth interactions of scions and size-controlling rootstocks of young apple trees. Plant Growth Regul.

[11] van Hooijdonk B, Woolley D, Warrington I, Tustin S (2011). Rootstocks modify scion architecture, endogenous hormones, and root growth of newly grafted ‘Royal Gala’ apple trees. J Am Soc Hortic Sci.

[12] Richards D, Thompson WK, Pharis RP (1986). The influence of dwarfing interstocks on the distribution and metabolism of xylem-applied [^3^H]gibberellin A_4_ in apple. Plant physiol.

[13] Van Hooijdonk BM, Woolley DJ, Warrington IJ, Tustin DS (2010). Initial alteration of scion architecture by dwarfing apple rootstocks may involve shoot-root-shoot signalling by auxin, gibberellin, and cytokinin. J Hortic Sci Biotech.

[14] Foster TM, McAtee PA, Waite CN, Boldingh HL, McGhie TK (2017). Apple dwarfing rootstocks exhibit an imbalance in carbohydrate allocation and reduced cell growth and metabolism. Hortic Res.

[15] Gregory P, Atkinson C, Bengough A, Else M, Fernández-Fernández F, Harrison R, Schmidt S (2013). Contributions of roots and rootstocks to sustainable, intensified crop production. J Exp Bot.

[16] Pina A, Cookson S, Calatayud A, Trinchera A, Errea P, Colla G, Perez-Alfocea F, Schwarz D (2017). Physiological and molecular mechanisms underlying graft compatibility. Vegetable grafting: principles and practices.

[17] Baron D, Esteves Amaro AC, Pina A, Ferreira G (2019). An overview of grafting re-establishment in woody fruit species. Sci Hortic.

[18] Gautier AT, Chambaud C, Brocard L, Ollat N, Gambetta GA, Delrot S, Cookson SJ (2019). Merging genotypes: graft union formation and scion-rootstock interactions. J Exp Bot.

[19] Zarrouk O, Gogorcena Y, Moreno MA, Pinochet J (2006). Graft compatibility between peach cultivars and prunus rootstocks. HortScience.

[20] Reig G, Salazar A, Zarrouk O, Forcada CFI, Val J, Moreno MA (2019). Long-term graft compatibility study of peach-almond hybrid and plum based rootstocks budded with European and Japanese plums. Sci Hortic.

[21] Tedesco S, Pina A, Fevereiro P, Kragler F (2020). A phenotypic search on graft compatibility in grapevine. Agronomy.

[22] Mosse B, Herrero J (1951). Studies on incompatibility between some pear and quince grafts. J Hortic Sci.

[23] Milne RJ, Grof CP, Patrick JW (2018). Mechanisms of phloem unloading: shaped by cellular pathways, their conductances and sink function. Curr Opin Plant Biol.

[24] Kasahara H (2016). Current aspects of auxin biosynthesis in plants. Biosci Biotechnol Biochem.

[25] Hedden P, Phillips A (2000). Gibberellin metabolism: new insights revealed by the genes. Trends Plant Sci.

[26] Davière JM, Achard P (2016). A pivotal role of DELLAs in regulating multiple hormone signals. Mol Plant.

[27] El-Showk S, Ruonala R, Helariutta Y (2013). Crossing paths: cytokinin signalling and crosstalk. Development.

[28] Zubo YO, Schaller GE (2020). Role of the cytokinin-activated type-B response regulators in hormone crosstalk. Plants.

[29] Fujioka S, Yokota T (2003). Biosynthesis and metabolism of brassinosteroids. Annu Rev Plant biol.

[30] Lin H, Wang R, Qian Q, Yan M, Meng X, Fu Z, Yan C, Jiang B, Su Z, Li J (2009). DWARF27, an iron-containing protein required for the biosynthesis of strigolactones, regulates rice tiller bud outgrowth. Plant Cell.

[31] Seto Y, Yasui R, Kameoka H, Tamiru M, Cao M, Terauchi R, Sakurada A, Hirano R, Kisugi T, Hanada A, et al. Strigolactone perception and deactivation by a hydrolase receptor DWARF14. Nat Commun. 2019;10(1):191. 10.1038/s41467-018-08124-7 PMC6331613 30643123

[32] Zhang Y, Zhao L, Zhao J, Li Y, Wang J, Guo R, Gan S, Liu C-J, Zhang K (2017). S5H/DMR6 encodes a salicylic acid 5-hydroxylase that fine-tunes salicylic acid homeostasis. Plant Physiol.

[33] Finkelstein R (2013). Abscisic acid synthesis and response. Arabidopsis Book.

[34] Majda M, Robert S (2018). The role of auxin in cell wall expansion. Int J Mol Sci.

[35] Kohorn BD, Kobayashi M, Johansen S, Riese J, Huang LF, Koch K, Fu S, Dotson A, Byers N (2006). An Arabidopsis cell wall-associated kinase required for invertase activity and cell growth. Plant J.

[36] Xie M, Zhang J, Tschaplinski TJ, Tuskan GA, Chen J-G, Muchero W (2018). Regulation of lignin biosynthesis and its role in growth-defense tradeoffs. Front Plant Sci.

[37] Vanholme R, Demedts B, Morreel K, Ralph J, Boerjan W (2010). Lignin biosynthesis and structure. Plant Physiol.

[38] Halbwirth H (2010). The creation and physiological relevance of divergent hydroxylation patterns in the flavonoid pathway. Int J Mol Sci.

[39] Shekhawat GS, Verma K (2010). Haem oxygenase (HO): an overlooked enzyme of plant metabolism and defence. J Exp Bot.

[40] Hanano A, Bessoule J-J, Heitz T, Blée E (2015). Involvement of the caleosin/peroxygenase RD20 in the control of cell death during Arabidopsis responses to pathogens. Plant Signal Behav.

[41] Kaplan B, Sherman T, Fromm H (2007). Cyclic nucleotide-gated channels in plants. FEBS Lett.

[42] Ruan Y-L, Jin Y, Yang Y-J, Li G-J, Boyer JS (2010). Sugar input, metabolism, and signaling mediated by invertase: roles in development, yield potential, and response to drought and heat. Mol Plant.

[43] Barratt DHP, Derbyshire P, Findlay K, Pike M, Wellner N, Lunn J, Feil R, Simpson C, Maule AJ, Smith AM (2009). Normal growth of Arabidopsis requires cytosolic invertase but not sucrose synthase. Proc Nat Acad Sci USA.

[44] Sergeeva LI, Keurentjes JJB, Bentsink L, Vonk J, van der Plas LHW, Koornneef M, Vreugdenhil D (2006). Vacuolar invertase regulates elongation of *Arabidopsis thaliana* roots as revealed by QTL and mutant analysis. Proc Nat Acad Sci USA.

[45] Wang L, Li X-R, Lian H, Ni D-A, He Y-K, Chen X-Y, Ruan Y-L (2010). Evidence that high activity of vacuolar invertase is required for cotton fiber and arabidopsis root elongation through osmotic dependent and independent pathways, respectively. Plant Physiol.

[46] Welham T, Pike J, Horst I, Flemetakis E, Katinakis P, Kaneko T, Sato S, Tabata S, Perry J, Parniske M (2009). A cytosolic invertase is required for normal growth and cell development in the model legume. Lotus japonicus J Exp Bot.

[47] Vaughn MW, Harrington GN, Bush DR (2002). Sucrose-mediated transcriptional regulation of sucrose symporter activity in the phloem. Proc Nat Acad Sci USA.

[48] Chiou T-J, Bush DR (1998). Sucrose is a signal molecule in assimilate partitioning. Proc Nat Acad Sci USA.

[49] Zhou Y, Chan K, Wang T, Hedley C, Offler C, Patrick J (2009). Intracellular sucrose communicates metabolic demand to sucrose transporters in developing pea cotyledons. J Exp Bot.

[50] Deeken R, Geiger D, Fromm J, Koroleva O, Ache P, Langenfeld-Heyser R, Sauer N, May ST, Hedrich R (2002). Loss of the AKT2/3 potassium channel affects sugar loading into the phloem of Arabidopsis. Planta.

[51] Decourteix M, Alves G, Bonhomme M, Peuch M, Ben Baaziz K, Brunel N, Guilliot A, Rageau R, Améglio T, Pétel G (2008). Sucrose (JrSUT1) and hexose (JrHT1 and JrHT2) transporters in walnut xylem parenchyma cells: their potential role in early events of growth resumption. Tree Physiol.

[52] Tang C, Huang D, Yang J, Liu S, Sakr S, Li H, Zhou Y, Qin Y (2010). The sucrose transporter HbSUT3 plays an active role in sucrose loading to laticifer and rubber productivity in exploited trees of Hevea brasiliensis (para rubber tree). Plant Cell Environ.

[53] Payyavula RS, Tay KHC, Tsai C-J, Harding SA (2011). The sucrose transporter family in Populus: the importance of a tonoplast PtaSUT4 to biomass and carbon partitioning. Plant J.

[54] Kraner ME, Link K, Melzer M, Ekici AB, Uebe S, Tarazona P, Feussner I, Hofmann J, Sonnewald U (2017). Choline transporter-like1 (CHER1) is crucial for plasmodesmata maturation in Arabidopsis thaliana. Plant J.

[55] Dettmer J, Ursache R, Campilho A, Miyashima S, Belevich I, O’Regan S, Mullendore DL, Yadav SR, Lanz C, Beverina L (2014). CHOLINE TRANSPORTER-LIKE1 is required for sieve plate development to mediate long-distance cell-to-cell communication. Nat Commun.

[56] Falhof A, Pedersen JT, Fuglsang AT, Palmgren M (2016). Plasma membrane H+-ATPase regulation in the center of plant physiology. Mol Plant.

[57] Philippar K, Ivashikina N, Ache P, Christian M, Lüthen H, Palme K, Hedrich R (2004). Auxin activates KAT1 and KAT2, two K+-channel genes expressed in seedlings of Arabidopsis thaliana. Plant J.

[58] MacMillan CP, Mansfield SD, Stachurski ZH, Evans R, Southerton SG (2010). Fasciclin-like arabinogalactan proteins: specialization for stem biomechanics and cell wall architecture in Arabidopsis and Eucalyptus. Plant J.

[59] Spartz AK, Ren H, Park MY, Grandt KN, Lee SH, Murphy AS, Sussman MR, Overvoorde PJ, Gray WM (2014). SAUR inhibition of PP2C-D phosphatases activates plasma membrane H+-ATPases to promote cell expansion in *Arabidopsis*. Plant Cell.

[60] Willige BC, Isono E, Richter R, Zourelidou M, Schwechheimer C (2011). Gibberellin regulates PIN-FORMED abundance and is required for auxin transport-dependent growth and development in Arabidopsis thaliana. Plant Cell.

[61] Ryan CA, Huffaker A, Yamaguchi Y (2007). New insights into innate immunity in Arabidopsis. Cell Microbiol.

[62] Huot B, Yao J, Montgomery BL, He SY (2014). Growth–defense tradeoffs in plants: a balancing act to optimize fitness. Mol Plant.

[63] Wang D, Pajerowska-Mukhtar K, Culler AH, Dong X (2007). Salicylic acid inhibits pathogen growth in plants through repression of the auxin signaling pathway. Curr Biol.

[64] Navarro L, Bari R, Achard P, Lisón P, Nemri A, Harberd NP, Jones JDG (2008). DELLAs control plant immune responses by modulating the balance of jasmonic acid and salicylic acid signaling. Curr Biol.

[65] Gallego-Giraldo L, Escamilla-Trevino L, Jackson LA, Dixon RA (2011). Salicylic acid mediates the reduced growth of lignin down-regulated plants. Proc Nat Acad Sci USA.

[66] Besseau S, Hoffmann L, Geoffroy P, Lapierre C, Pollet B, Legrand M (2007). Flavonoid accumulation in Arabidopsis repressed in lignin synthesis affects auxin transport and plant growth. Plant Cell.

[67] Mouradov A, Spangenberg G (2014). Flavonoids: a metabolic network mediating plants adaptation to their real estate. Front Plant Sci.

[68] Brown DE, Rashotte AM, Murphy AS, Normanly J, Tague BW, Peer WA, Taiz L, Muday GK (2001). Flavonoids act as negative regulators of auxin transport in vivo in arabidopsis. Plant Physiol.

[69] Solfanelli C, Poggi A, Loreti E, Alpi A, Perata P (2006). Sucrose-specific induction of the anthocyanin biosynthetic pathway in Arabidopsis. Plant Physiol.

[70] Xie Y, Tan H, Ma Z, Huang J (2016). DELLA proteins promote anthocyanin biosynthesis via sequestering MYBL2 and JAZ suppressors of the MYB/bHLH/WD40 complex in Arabidopsis thaliana. Mol Plant.

[71] Li Y, Van Den Ende W, Rolland F (2014). Sucrose induction of anthocyanin biosynthesis is mediated by della. Mol Plant.

[72] Tauzin AS, Giardina T (2014). Sucrose and invertases, a part of the plant defense response to the biotic stresses. Front Plant Sci.

[73] Zhou Y, Underhill S (2016). Breadfruit (Artocarpus altilis) gibberellin 2-oxidase genes in stem elongation and abiotic stress response. Plant Physiol Biochem.

[74] Zhou Y, Underhill SJR (2018). Plasma membrane H -ATPase activity and graft success of breadfruit (Artocarpus altilis) onto interspecific rootstocks of marang (A. odoratissimus) and pedalai (A. sericicarpus). Plant Biol.

[75] Birnberg PR, Brenner ML (1984). A one-step enzymatic assay for sucrose with sucrose phosphorylase. Anal Biochem.

[76] Mirajkar SJ, Suprasanna P, Vaidya ER (2016). Spatial distribution and dynamics of sucrose metabolising enzymes in radiation induced mutants of sugarcane. Plant Physiol Biochem.

[77] Tomlinson KL, McHugh S, Labbe H, Grainger JL, James LE, Pomeroy KM, Mullin JW, Miller SS, Dennis DT, Miki BLA (2004). Evidence that the hexose-to-sucrose ratio does not control the switch to storage product accumulation in oilseeds: analysis of tobacco seed development and effects of overexpressing apoplastic invertase. J Exp Bot.

[78] Bradford MM (1976). A rapid and sensitive method for the quantitative microgram quantities of protein utilizing the principle of protein-dye binding. Anal Biochem.

[79] Cerveau N, Jackson DJ (2016). Combining independent de novo assemblies optimizes the coding transcriptome for nonconventional model eukaryotic organisms. BMC Bioinformatics.

[80] Grabherr MG, Haas BJ, Yassour M, Levin JZ, Thompson DA, Amit I, Adiconis X, Fan L, Raychowdhury R, Zeng Q (2011). Full-length transcriptome assembly from RNA-Seq data without a reference genome. Nat Biotechnol.

[81] Schulz MH, Zerbino DR, Vingron M, Birney E (2012). Oases: robust de novo RNA-seq assembly across the dynamic range of expression levels. Bioinformatics.

[82] Bushmanova E, Antipov D, Lapidus A, Prjibelski AD (2019). rnaSPAdes: a de novo transcriptome assembler and its application to RNA-Seq data. GigaScience.

[83] Fu L, Niu B, Zhu Z, Wu S, Li W (2012). CD-HIT: accelerated for clustering the next-generation sequencing data. Bioinformatics.

[84] Haas BJ, Papanicolaou A, Yassour M, Grabherr M, Blood PD, Bowden J, Couger MB, Eccles D, Li B, Lieber M (2013). De novo transcript sequence reconstruction from RNA-seq using the trinity platform for reference generation and analysis. Nat Protoc.

