# Supplementary material for: Differential transcription pathways associated with rootstock-induced dwarfing in breadfruit (Artocarpus altilis) scions
Source: BMC Plant Biol. 2021 Jun 5;21:261. doi: 10.1186/s12870-021-03013-6 (PMC8178858; doi:10.1186/s12870-021-03013-6)
Supplement: Supplementary file 1 — Additional file 1: Table S1. Morphological assessment of breadfruit plants growing on different rootstocks*. The 1-year and 2-year compatibility rates represent mean ± SD of two independent trials, each with eight biological replicates in each graft combination from the start. All other values represent mean ± SE of six biological replicates. Values with different letters from the same row are significantly different (p < 0.05). * Final measurement at 26 months after grafting. [file 12870_2021_3013_MOESM1_ESM.docx]

Table S1 Morphological assessment of breadfruit plants growing on different rootstocks*.

|  | | Seft-graft | Marang rootstock |
| --- | --- | --- | --- |
| Final scion height (cm) | | 174.38 ± 11.31 *a* | 72.10 ± 9.36 *b* |
| Scion main stem diameter (cm) | | 3.22 ± 0.08  *a* | 2.25 ± 0.18 *b* |
| Ratio of stem diameter above and below graft union | | 0.96 ± 0.15 *a* | 1.05 ± 0.06 *a* |
| Node number on scion main stems | | 28.04 ± 3.36 *a* | 29.19 ± 6.42 *a* |
| Number of branches per plant | | 2.80 ± 0.42 *a* | 1.00 ± 0.61 *b* |
| Main stem elongation rate (cm/month) | | 6.86 ± 0.71 *a* | 3.37 ± 0.46 *b* |
| Branch elongation rate (cm/month) | | 7.93 ± 1.18 *a* | 4.45 ± 0.54 *b* |
| Second internode length in scion main stems (cm) | | 6.06 ± 0.86 *a* | 2.97 ± 0.44 *b* |
| Leaf numbers per plant | | 8.75 ± 0.95 *a* | 6.00 ± 0.81 *b* |
| Leaf chlorophyll *a* (mg/g) | | 2.28 ± 0.45 *a* | 2.18 ± 0.28 *a* |
| Leaf chlorophyll *b* (mg/g) | | 0.86 ± 0.09 *a* | 1.12 ± 0.21 *a* |
| leaf chlorophyll *a* + *b* (mg/g) | | 3.14 ± 0.47 *a* | 3.41 ± 0.39 *a* |
| Leaf chlorophyll *a*/*b* |  | 2.71 ± 0.65 *a* | 2.09 ± 0.43 *a* |
| Leaf length | | 78.11 ± 3.26 *a* | 63.02 ± 5.40 *b* |
| Leaf width |  | 60.85 ± 2.02 *a* | 49.60 ± 2.02 *b* |
| 1- year graft compatibility (%) | | 81.25 ± 8.84 *a* | 75.00 ± 0.00 *a* |
| 2- year graft compatibility (%) | | 75.00 ± 0.00 *a* | 68.75 ± 8.84 *a* |

The 1-year and 2-year compatibility rates represent mean ± SD of two independent trials, each with eight biological replicates in each graft combination from the start. All other values represent mean ± SE of six biological replicates. Values with different letters from the same row are significantly different (p < 0.05). * Final measurement at 26 months after grafting.
