# Supplementary material for: Differential transcription pathways associated with rootstock-induced dwarfing in breadfruit (Artocarpus altilis) scions
Source: BMC Plant Biol. 2021 Jun 5;21:261. doi: 10.1186/s12870-021-03013-6 (PMC8178858; doi:10.1186/s12870-021-03013-6)
Supplement: Supplementary file 2 — Additional file 2: Table S2. de novo transcriptome assembly metrics. [file 12870_2021_3013_MOESM2_ESM.docx]

**TableS1 *de novo* transcriptome assembly metrics**

| **Assembly** | **Final transcriptome** | | | **Oases** | | **Rnaspades** | | **Trinity** |
| --- | --- | --- | --- | --- | --- | --- | --- | --- |
| # contigs (>= 297 bp) | | 212,879 | 617,672 | | 418,390 | | 571,056 | |
| # contigs (>= 1000 bp) | | 154,211 | 300,644 | | 82,640 | | 135,896 | |
| # contigs (>= 5000 bp) | | 11,393 | 24,118 | | 1,664 | | 5,418 | |
| # contigs (>= 10000 bp) | | 523 | 1,179 | | 60 | | 209 | |
| # contigs (>= 25000 bp) | | 24 | 31 | | 8 | | - | |
| # contigs (>= 50000 bp) | | 15 | 23 | | - | | - | |
| Total length (>= 297 bp) | | 455,209,646 | 956,929,039 | | 293,318,916 | | 488,980,954 | |
| Total length (>= 1000 bp) | | 420,683,109 | 836,479,968 | | 168,585,108 | | 317,665,822 | |
| Total length (>= 5000 bp) | | 75,641,748 | 159,430,058 | | 10,698,051 | | 34,462,206 | |
| Total length (>= 10000 bp) | | 7,725,619 | 16,341,638 | | 903,842 | | 2,526,919 | |
| Total length (>= 25000 bp) | | 1,580,955 | 2,278,376 | | 235,387 | | - | |
| Total length (>= 50000 bp) | | 1,287,912 | 1,986,522 | | - | | - | |
| # contigs | | 212,879 | 617,672 | | 418,390 | | 571,056 | |
| Largest contig | | 160,154 | 160,154 | | 42,477 | | 22,225 | |
| Total length | | 455,209,646 | 956,929,039 | | 293,318,916 | | 488,980,954 | |
| GC (%) | | 40.66 | 39.49 | | 37.31 | | 38.7 | |
| N50 | | 2,928 | 2,886 | | 1,281 | | 1,740 | |
| N75 | | 1,927 | 1,768 | | 440 | | 616 | |
| L50 | | 52,238 | 110,083 | | 63,290 | | 80,829 | |
| L75 | | 99,820 | 214,370 | | 160,709 | | 198,778 | |
| # N's per 100 kbp | | 2 | 11 | | 27 | | - | |
| Complete BUSCOs (C) | | 96.8 | 93.8 | | 66.1 | | 90.1 | |
| Fragmented BUSCOs (F) | | 2 | 4.5 | | 23.5 | | 7.2 | |
| Missing BUSCOs (M) | | 1.2 | 1.7 | | 10.4 | | 2.7 | |
| Total BUSCO groups searched | | 2121 | 2121 | | 2121 | | 2121 | |
| MapEff (%) | |  |  | |  | |  | |
| Self-graft library 1 | | 94.54 | 96.36 | | 96.16 | | 97.24 | |
| Self-graft library 2 | | 94.18 | 97.28 | | 96.73 | | 98.05 | |
| Self-graft library 3 | | 93.68 | 96.36 | | 95.95 | | 97.2 | |
| reference library | | 93.61 | 96.02 | | 95.89 | | 97.01 | |
| Marang-rootstock library 1 | | 92.88 | 95.84 | | 95.78 | | 97.15 | |
| Marang-rootstock library 2 | | 94.06 | 96.83 | | 96.58 | | 97.86 | |
| Marang-rootstock library 3 | | 92.88 | 95.79 | | 95.86 | | 97.07 | |
