# Supplementary material for: Differential transcription pathways associated with rootstock-induced dwarfing in breadfruit (Artocarpus altilis) scions
Source: BMC Plant Biol. 2021 Jun 5;21:261. doi: 10.1186/s12870-021-03013-6 (PMC8178858; doi:10.1186/s12870-021-03013-6)
Supplement: Supplementary file 4 — Additional file 4: Table S4. Quantitative real-time PCR primers. [file 12870_2021_3013_MOESM4_ESM.docx]

**Table S3 Quantitative real-time PCR primers**

| Gene ID | Forward primer | Reversed primer |
| --- | --- | --- |
| oases_368732 | CAGTAATGGTGCCGGAGAGG | GAACGCTCACGCCACTTTCT |
| trinity_237646 | GCTGCTTCACTGCAAGCTCA | TCTGGGGAAGCATTCTCTGC |
| oases_96998 | AGCCATTGTTCAGGCTGCTC | GGCGTTGTGTCTCGGCTAAT |
| oases_383793 | GTGGCAAGATGGGTTGATCC | GAGACCCTTCACCCGGAGAT |
| rnaspades_65154 | CCGCCGGATGTGATGTTAAT | TGAGCAGGGTTTGGTTCTCC |
| rnaspades_39330 | AATGGGCATTGGTGGACACT | TCCAGCGGTGGAACTGATTT |
| trinity_59504 | TCGCAAACTGCGTAACGAGA | CAAGCCATGCCAGGGTAAAT |
| oases_192581 | CCAACAGGCCTATGCTGACC | TCGCTGCATAGCTGATTCCA |
| oases_181688 | CACCACGTTGGGCAAGTGTC | TCCATTGCGCTTCTGTCGAA |
| oases_261197 | GGAAGCTGAGGACGAGCGTA | GCCTTCTACCACCGCAACAC |
| oases_488859 | CGACATTCCGTGGTTGAACA | TCGTCCGACTCGTCCTTCTC |
| trinity_131914 | GCCCTGAACACCCAAACAAG | CATGTCCCAAGGCACAAACA |
| oases_223828 | ACACCTCCACAGCCATGTGA | AATGACGCTGGCTGGTGTTT |
| oases_237411 | GAGTCCAGGACCGTTGATCG | CCATTCTACGCTCCGCAAGT |
| rnaspades_110399 | TGCCCAAGCTGTGAGAATGA | GCTTGCAAAGGCTGTGTCCT |
| trinity_68041 | CAACCACAAATGGCGTTTCA | GGTGACATGGGCGAAGAAAG |
| rnaspades_66441 | CCTGTTGCTGTTGCTGGTTG | AACCCCTGCCTGCTACTCCT |
| trinity_95243 | TATGGGATCTGAGGGCGAGA | TCCACAGCGTCAGGTGAGTC |
| oases_140377 | AGTCTCCTCGCAAACGCAAC | GCCTCTGCAAATCCATCCAC |
| trinity_380298 | AGAGGGCCAACTTCCTTCGT | GGCTCCAGGCTTTTCAACAA |
| oases_190576 | TCAATTGGGGCAGTGTTTCA | TGGAAATGAGCGAAGCAACA |
