# Supplementary figures and images for: Differential transcription pathways associated with rootstock-induced dwarfing in breadfruit (Artocarpus altilis) scions
Source: BMC Plant Biol. 2021 Jun 5;21:261. doi: 10.1186/s12870-021-03013-6 (PMC8178858; doi:10.1186/s12870-021-03013-6)

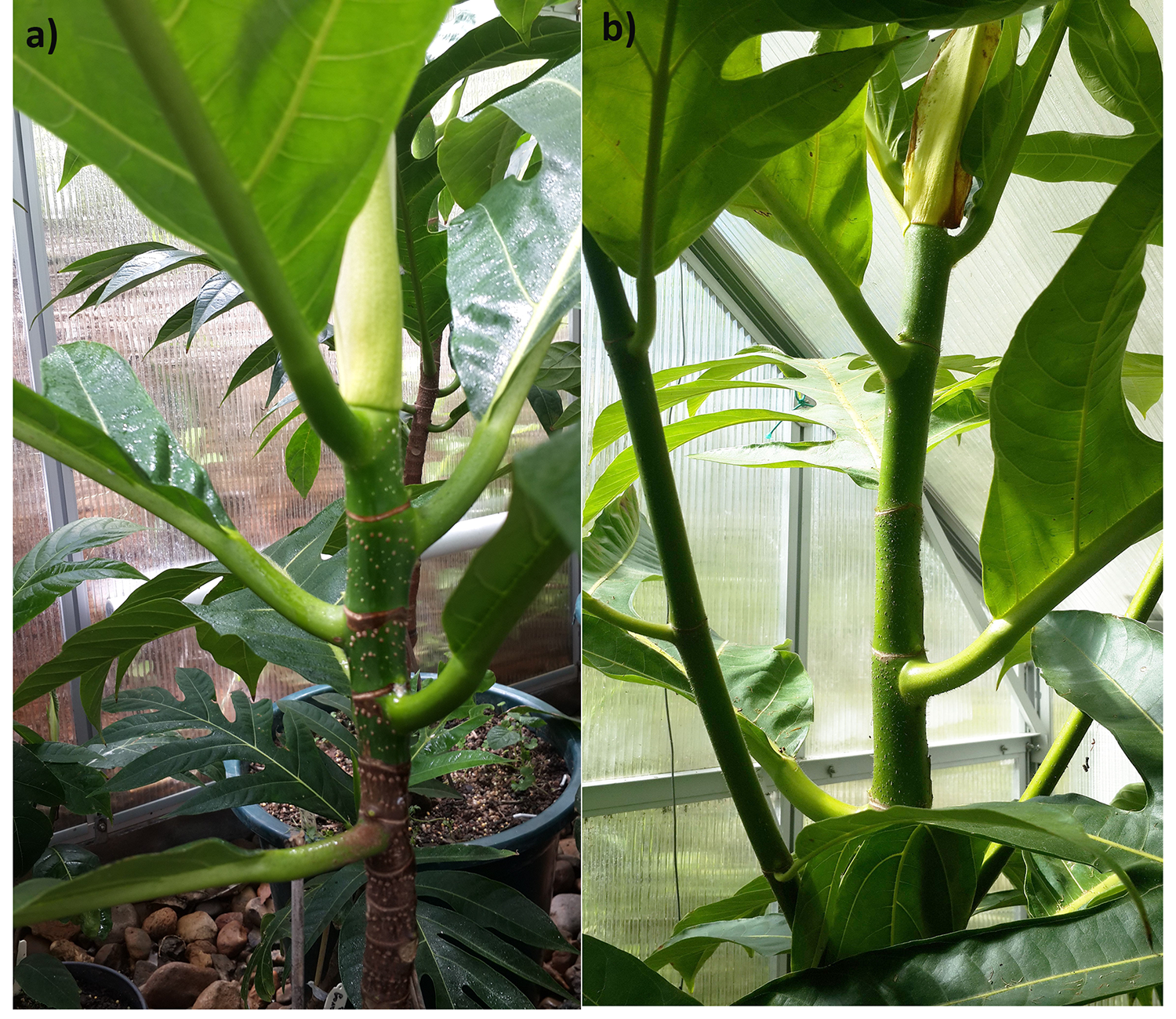

Supplement: Supplementary file 5 — Additional file 5: Figure S1. Representatives of graft-compatible phenotype of breadfruit plants growing on marang rootstocks (a) and self-graft (b) in the period from 3 months to 26 months after grafting. [file 12870_2021_3013_MOESM5_ESM.tif]

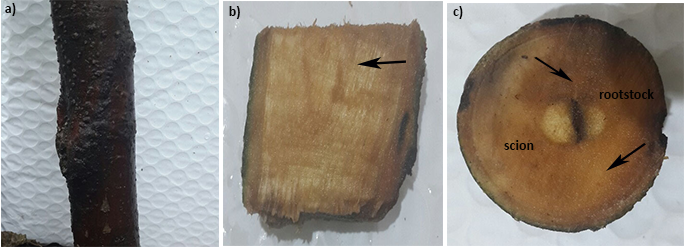

Supplement: Supplementary file 6 — Additional file 6: Figure S2. Representative images of longitudinal and cross sections through a graft union of breadfruit scion grafted on marang rootstock. a) Graft union from approach graft; b) Longitudinal section of a graft union; c) Cross-section of a graft union. Graft lines are indicated by arrows. [file 12870_2021_3013_MOESM6_ESM.tif]
